# *Lactobacillus acidophilus* CICC 22162 alleviates sleep deprivation-induced cognitive impairment by remodeling gut microbiota and enhancing S-adenosylmethionine-mediated neuroimmune regulation

**DOI:** 10.3389/fmicb.2026.1889475

**Published:** 2026-08-19

**Authors:** Zhiqiang Jiang, Zhuohan Zhang, Mengqi Shi, Xiaoping Li, Xuefei Zhang, Chang Liu, Yuhao Wang, Jin Zhu, Chunyan Gu, Zhan Yang

**Affiliations:** 1 School of Medicine, Nanjing University of Chinese Medicine, Nanjing, China; 2 Huadong Medical Institute of Biotechniques, Nanjing, China; 3 Department of Infectious Disease, The First Affiliated Hospital of Nanjing Medical University, Nanjing, China; 4 Lushan Rehabilitation and Recuperation Center, Jiujiang, China; 5 Department of General Surgery, The Second Affiliated Hospital of Nanjing Medical University, Nanjing, China; 6 School of Life Sciences, Xuzhou Medical University, Xuzhou, China

**Keywords:** cognitive impairment, fecal microbiota transplantation, gut microbiota, *Lactobacillus acidophilus*, microbiota–gut–brain axis, neuroinflammation, S-adenosylmethionine, sleep deprivation

## Abstract

**Introduction:**

Sleep deprivation (SD) impairs cognitive function and induces hippocampal injury, yet the gut-derived mechanisms underlying these deficits remain incompletely understood.

**Methods:**

Here, we established a mouse SD model using a modified multiple-platform water environment method and evaluated the effects of oral administration of *Lactobacillus acidophilus* CICC 22162.

**Results:**

SD caused gut microbial dysbiosis and compromised colonic barrier integrity, leading to hippocampal inflammation, neuronal apoptosis, and synaptic protein loss. Intervention with *L. acidophilus* CICC 22162 partially restored microbial balance, strengthened intestinal barrier proteins, reduced hippocampal inflammation and neuronal apoptosis, and improved cognitive performance. Fecal microbiota transplantation (FMT) experiments demonstrated that protective effects could be partially transferred via the gut microbiota from treated donors, highlighting the functional role of the intestinal microbial community. Untargeted metabolomics identified S-adenosylmethionine (SAM) as a candidate microbiota-associated metabolite linked to neuroprotection, and *in vivo* and ex vivo validation showed that SAM reproduced cognitive benefits through α7 nicotinic acetylcholine receptor (α7nAChR)-related signaling, including enhanced JAK2/STAT3 phosphorylation and suppressed NF-κB activation.

**Discussion:**

These findings delineate a gut microbiota–SAM–α7nAChR neuroimmune pathway through which *L. acidophilus* CICC 22162 alleviates SD-associated cognitive dysfunction, providing mechanistic support for microbiota-targeted interventions against sleep loss-induced cognitive impairment.

## 1 Introduction

Sleep deprivation (SD) may result from curtailed sleep, fragmented sleep, poor sleep quality, or disruption of circadian timing, and its prevalence has increased across populations of different ages (Kecklund and Axelsson, 2016; Grandner, 2017). Loss of sleep affects multiple physiological systems and has been associated with cardiovascular dysfunction, metabolic abnormalities, and immune-inflammatory imbalance (Irwin et al., 2016; Wang et al., 2016; Tobaldini et al., 2017). The brain is especially sensitive to sleep-related stress. Evidence from experimental and clinical studies suggests that SD interferes with memory consolidation, synaptic remodeling, and neuronal activity, thereby impairing spatial learning, working memory, and recognition memory (Rasch and Born, 2013; Klinzing et al., 2019; Yuan and Wang, 2025). Recurrent or long-lasting sleep loss may also be associated with neurodegenerative processes and increased susceptibility to Alzheimer’s disease and dementia-related cognitive impairment (Bubu et al., 2017; Erkkinen et al., 2018). Despite substantial investigation into the neural consequences of SD, effective and safe interventions for SD-related cognitive dysfunction remain limited.

Neuroinflammation is a key process contributing to the cognitive consequences of sleep deprivation (SD). Sleep loss stimulates microglia and astrocytes, increases inflammatory mediators, such as IL-6, TNF-*α*, and IL-1*β*, partly through NF-κB-dependent signaling. This inflammatory cascade may impair synaptic plasticity and contribute to neuronal injury (Zhao et al., 2019; Yuan and Wang, 2025; Jiang J. et al., 2026). However, brain-localized inflammation alone is insufficient to explain the systemic physiological consequences of SD; therefore, additional pathways, such as the microbiota–gut–brain axis (MGBA), must be considered. The MGBA provides an additional route by which intestinal microorganisms may influence brain function through immune, metabolic, neural, and barrier-related signals (Loh et al., 2024; O’Riordan et al., 2025; Zhang et al., 2025). Wang et al. demonstrated, and Sun et al. further summarized, that SD disrupts gut microbial homeostasis and alters several commensal taxa with potential health-promoting functions, thereby contributing to microbial dysbiosis (Wang et al., 2021; Sun et al., 2023). Such dysbiosis may compromise intestinal epithelial barrier homeostasis and amplify peripheral and central inflammatory responses, particularly those linked to hippocampal dysfunction (Tilg et al., 2020; Wang et al., 2021; Aburto and Cryan, 2024). Although SD-induced dysbiosis has been linked to cognitive injury, the relative contribution of microbial alterations and the downstream metabolites and host signaling pathways involved remain incompletely defined.

Probiotic intervention provides a potential strategy for modulating the MGBA because selected beneficial microbial strains, at appropriate doses, can promote host health and may influence cognitive-related outcomes (Hill et al., 2014; Liu et al., 2025; Zandifar et al., 2025). *Lactobacillus acidophilus* is a representative probiotic species with reported effects on intestinal epithelial barrier regulation and inflammatory signaling (Al-Sadi et al., 2021; Haque et al., 2024). Huang et al. and Ma et al. have also indicated that *L. acidophilus* or probiotic supplementation may benefit cognitive outcomes in neurological or inflammation-related contexts (Huang Y. et al., 2025; Ma et al., 2025). However, its role in SD-induced cognitive impairment has not been fully defined. In particular, whether *L. acidophilus*-mediated microbiota remodeling generates metabolic signals that modulate hippocampal inflammation and neuronal injury remains to be determined.

Given the evidence that gut microbiota contributes to SD-induced inflammatory responses and cognitive impairment and that *L. acidophilus* or probiotic supplementation may influence cognitive outcomes (Wang et al., 2021; Huang Y. et al., 2025; Ma et al., 2025), we investigated whether *L. acidophilus* could alleviate SD-related cognitive deficits. We also examined the microbiota-dependent metabolic events underlying this effect. SD was established in mice using a modified multi-platform water setup. Gut microbial composition was characterized by 16S rRNA amplicon sequencing, and fecal microbiota transplantation (FMT) experiments were used to determine whether the remodeled intestinal microbial community could confer the protective phenotype. Untargeted fecal metabolomics was subsequently used to detect metabolites associated with *L. acidophilus* intervention. In follow-up mouse and cell-based experiments, we explored whether S-adenosylmethionine (SAM) and α7 nicotinic acetylcholine receptor (α7nAChR)-mediated neuroimmune pathways contributed to the observed protective effects, and we further assessed their role in modulating hippocampal neuroinflammation, considering the well-established function of α7nAChR-mediated cholinergic signaling in inflammatory regulation (Wang et al., 2003; de Jonge et al., 2005). Together, this study identifies a potential microbiota–SAM–α7nAChR-related pathway through which *L. acidophilus* may restrain hippocampal neuroinflammation and improve cognitive performance after sleep loss.

## 2 Materials and methods

### 2.1 Animals, bacterial strain, and cell lines

Eight-week-old male C57BL/6 J mice were purchased from Nanjing Kaijia Biotechnology Co., Ltd. (Nanjing, China). After arrival, animals were acclimated under specific pathogen-free conditions at 22 ± 2 °C and 50 ± 5% humidity, with a 12-h light/dark schedule and free access to standard chow and water. All procedures were reviewed and approved by the Ethics Committee of Huadong Medical Institute of Biotechniques, Nanjing, Jiangsu, China [protocol code: (2024) No. 22; approval date: December 2024].

The laboratory-preserved *Lactobacillus acidophilus* strain used in this work was provided by the China Center of Industrial Culture Collection (CICC; strain no. CICC 22162). For animal administration, *L. acidophilus* CICC 22162 was grown anaerobically in MRS broth (Oxoid, Cat# CM1175B) at 37 °C for 24 h. Bacterial pellets were obtained after centrifugation at 3,000 × g for 10 min at 4 °C, washed, and then resuspended in sterile normal saline. The bacterial density was estimated using a WGZ-XT bacterial turbidity meter (Qiwei, China) according to the manufacturer’s McFarland-to-CFU conversion. The final suspension was adjusted to approximately 5 × 10^9^ CFU/mL, and each mouse received 200 μL by oral gavage, corresponding to approximately 1 × 10^9^ CFU per mouse per day.

BV2 mouse microglial cells and HT22 mouse hippocampal neuronal cells were purchased from Procell Life Science & Technology Co., Ltd. The identifiers were Cat# CL-0493, RRID: CVCL_0182 for BV2 cells and Cat# CL-0697, RRID: CVCL_0321 for HT22 cells. Cells were maintained in DMEM (Gibco, Cat# C11885500BT) containing 10% fetal bovine serum (Corning, Cat# 35-079-CV) and 1% penicillin–streptomycin (Gibco, Cat# 15140122) and kept in a humidified incubator set at 37 °C and 5% CO₂.

### 2.2 Experimental design, sleep deprivation, and probiotic treatment

After 3 days of acclimatization, mice were assigned to one of four experimental conditions: normal control (NC), sleep deprivation (SD), SD plus *L. acidophilus* treatment (SD + LA), or *L. acidophilus* alone (LA). SD was produced using a modified multiple-platform water environment method based on the small-platforms-over-water principle (Machado et al., 2004; Arthaud et al., 2015). The tank measured 85 × 45 × 22 cm, with the water level maintained at 9 cm, the platform height set at 10 cm, and the water was maintained at 28 °C during the SD procedure.

Mice in the SD and SD + LA groups were placed on small platforms with a diameter of 2.5 cm for 7 consecutive days, with a 2-h rest interval each day. Mice in the NC and LA groups were exposed to the same water environment but were placed on large cylindrical platforms with a diameter of 10 cm, which allowed free movement and served as a control for water-environment-related stress without inducing sleep deprivation.

For 7 consecutive days, animals assigned to the SD + LA and LA groups were given 200 μL of freshly prepared *L. acidophilus* suspension, corresponding to approximately 1 × 10^9^ CFU per mouse via oral gavage once daily. Sterile saline was administered to the NC and SD groups at the same volume and via the same route.

### 2.3 16S rRNA gene sequencing and analysis

After the *in vivo* study was completed, freshly voided fecal samples were collected under aseptic conditions and kept at −80 °C until further processing. Library preparation, 16S rRNA amplicon sequencing, and primary bioinformatic processing were performed by Biomarker Technologies Co., Ltd. (Wuhan, China). The gut microbiota was profiled by sequencing the V3–V4 amplicons of the bacterial 16S rRNA gene amplified using primers 338F (5′-ACTCCTACGGGAGGCAGCA-3′) and 806R (5′-GGACTACHVGGGTWTCTAAT-3′) on an Illumina NovaSeq system. Following quality control and primer trimming, clean tags were classified into OTUs at 97% sequence similarity, and representative OTU sequences were annotated using the SILVA reference database (RRID: SCR_006423). Microbial diversity, taxonomic structure, and group-enriched taxa were evaluated at the OTU level, and LEfSe was applied for discriminative feature analysis (RRID: SCR_014609). Sequencing reads from this study are available through the NCBI SRA repository (RRID: SCR_004891) with BioProject accession PRJNA1453587 (NCBI BioProject, RRID: SCR_004801; Jiang et al., 2026a).

### 2.4 Quantitative real-time PCR

Fecal *L. acidophilus* abundance was determined by absolute quantitative real-time PCR. The oligonucleotide primers used for amplification were published *L. acidophilus* species-specific primers: LacidoF, 5′-TGCAAAGTGGTAGCGTAAGC-3′; LacidoR, 5′-CCTTTCCCTCACGGTACTG-3′. These primers target the 16S–23S spacer region (Jomehzadeh et al., 2020). No internal reference gene was used because absolute quantification was performed using a standard curve. Genomic DNA from the *L. acidophilus* standard strain was purified using the QIAamp DNA Mini Kit (Qiagen, Cat# 51309), and the target fragment was amplified with TaKaRa Ex Taq^®^ (Takara Bio, Cat# RR030A). The PCR product was diluted in a tenfold series to generate a standard curve. For fecal DNA quantification, qPCR reactions were prepared with TB Green^®^ Premix Ex Taq™ II (Takara Bio, Cat# RR820A) and detected using a QuantStudio™ 5 Real-Time PCR System (Thermo Fisher Scientific). Triplicate reactions were set up for each sample, and melting-curve analysis was used to assess amplification specificity.

### 2.5 Intestinal histological and barrier assessments

After completion of behavioral testing, colonic specimens were harvested for histological examination and protein measurement. Samples used for morphology and immunohistochemistry were fixed in 4% paraformaldehyde (Beyotime, Cat# P0099), whereas tissues intended for protein extraction were snap-frozen. Colonic samples prepared as paraffin blocks were cut into 4 μm sections for hematoxylin and eosin (H&E) staining using an H&E staining kit (Nanjing FreshBio Technology Co., Ltd., Cat# FH111500).

MUC2 expression in colon sections was assessed by immunohistochemical staining using an immunohistochemistry staining kit (Nanjing FreshBio Technology Co., Ltd., Cat# FH222100RM). Briefly, sections were processed for antigen unmasking and exposed to an anti-MUC2 primary antibody overnight at 4 °C (Abcam, Cat# ab272692, RRID: AB_2888616). Immunoreactivity was detected with an HRP-linked secondary antibody followed by DAB chromogenic development using a DAB substrate kit (Servicebio, Cat# G1212). Slides were scanned on a Pannoramic MIDI II system (3DHISTECH), and the resulting images were analyzed with ImageJ (RRID: SCR_003070).

For immunoblotting, total protein from colonic tissue was extracted using radioimmunoprecipitation assay (RIPA) buffer. Protein lysates were probed with antibodies recognizing Occludin (Proteintech, Cat# 27260-1-AP, RRID: AB_2880820), Claudin-1 (Proteintech, Cat# 28674-1-AP, RRID: AB_2881190), and MUC2 (Abcam, Cat# ab272692, RRID: AB_2888616). Chemiluminescent signals were recorded on a Tanon 5,200 imaging system, and target protein levels were normalized to *β*-actin (Proteintech, Cat# 66009-1-Ig, RRID: AB_2687938).

### 2.6 Behavioral tests

A series of behavioral tests was performed, including the novel object recognition test (XR-XX117), Y-maze test (XR-XY1032), Morris water maze test (XR-XM101), and elevated plus maze test (XR-XG201). All equipment was obtained from Shanghai Xinruan Information Technology Co., Ltd. Animal movements and behavioral performance were recorded and analyzed using an automated video-tracking system.

For the novel object recognition test, mice were subjected to habituation, a training session with a pair of identical objects, and a test session with a familiar object and a novel object, with minor modifications to the protocol described by Lueptow (2017). Recognition performance was expressed as the discrimination index together with the fraction of exploration time directed toward the novel object. In the Y-maze, one arm was closed during training and reopened as the novel arm during testing, following the procedure reported by Kraeuter et al. (2019). Spatial working memory was determined from the preference for entering and exploring the novel arm. In the Morris water maze, mice underwent acquisition training for 5 consecutive days with four trials each day; each trial lasted up to 60 s, and the platform was removed for the probe trial, according to the method of Vorhees and Williams (2006), with minor modifications. Learning performance was assessed by escape latency, while memory retention was determined by the time spent in the target quadrant and the number of crossings over the previous platform location. For the elevated plus maze test, mice were positioned in the central zone facing an open arm and permitted to explore freely for 5 min, based on the method described by Walf and Frye (2007). Elevated plus maze performance was evaluated using the percentage of open-arm entries and the duration spent in open arms. The apparatus was cleaned between consecutive trials to reduce olfactory interference.

### 2.7 Hippocampal histopathological and biochemical analyses

After behavioral assessment, hippocampal tissues were collected for histopathological and biochemical evaluation. Apoptotic cells in 4-μm hippocampal paraffin sections were detected with a fluorescein (FITC) TUNEL Cell Apoptosis Detection Kit (Servicebio, Cat# G1501), followed by DAPI nuclear counterstaining (Servicebio, Cat# G1012).

For detection of cytokines and phenotype-related markers, hippocampal specimens were homogenized in pre-chilled phosphate-buffered saline (PBS). Following centrifugation, the clarified supernatants were subjected to ELISA-based quantification using IL-1*β* ELISA kit (LAPUDA, Cat# LA128804H), IL-6 ELISA kit (LAPUDA, Cat# LA128802H), IL-10 ELISA kit (LAPUDA, Cat# LA128803H), TNF-*α* ELISA kit (LAPUDA, Cat# LA128801H), IL-4 ELISA kit (LAPUDA, Cat# LA128809H), and Arginase-1 ELISA kit (LAPUDA, Cat# LA128176H) according to the manufacturer’s protocol. The measured values were normalized to the total protein concentration of each sample.

For immunoblotting, hippocampal protein lysates were probed with antibodies against iNOS (ABclonal, Cat# A3774, RRID: AB_3094627), Arginase-1 (ABclonal, Cat# A25808, RRID: AB_3697284), Synapsin-1 (ABclonal, Cat# A24122, RRID: AB_3661635), and PSD-95 (ABclonal, Cat# A6194, RRID: AB_2766806). *β*-actin (Proteintech, Cat# 66009-1-Ig, RRID: AB_2687938) was used as the internal control.

### 2.8 Fecal microbiota transplantation

Recipient mice were first treated with an antibiotic cocktail to deplete the resident gut microbiota. The cocktail contained metronidazole (Sangon Biotech, Cat# A600633), neomycin sulfate (Sangon Biotech, Cat# A430130), ampicillin (Sangon Biotech, Cat# A430258), and vancomycin (Sangon Biotech, Cat# A600983) at doses of 200, 200, 200, and 100 mg/kg, respectively. Thus, the dose ratio of metronidazole:neomycin sulfate:ampicillin:vancomycin was 2:2:2:1 (Gong et al., 2018; Zhang et al., 2024). To reduce the resident intestinal microbiota, mice received the antibiotic mixture by oral gavage once daily over a 5-day period; depletion of culturable fecal bacteria was assessed before FMT by culturing fecal samples on Columbia blood agar plates (Huankai Microbial, Cat# CP0160, 90 mm) and quantifying colony counts (Almonte et al., 2022).

Donor fecal pellets were obtained from mice in the NC, SD, SD + LA, and LA groups. The collected fecal material was homogenized in sterile PBS to a final concentration of 0.125 g/mL following the murine FMT preparation protocols reported by Shang et al. (2022) and Tan et al. (2022). Recipient mice were then gavaged with 200 μL of the corresponding fecal suspension once daily for 7 days, corresponding to approximately 25 mg of donor feces per mouse per day. The transplantation dose was standardized by fecal mass and gavage volume, and viable bacterial enumeration was not separately performed. After completion of FMT, all recipient mice were subjected to 7 days of SD, with a 2-h rest interval each day. Behavioral testing and tissue collection were performed after the SD procedure.

### 2.9 Untargeted fecal metabolomics

Fecal samples from the NC, SD, SD + LA, and LA groups were collected after the *in vivo* experiment and kept at −80 °C until metabolomic analysis. Five biological replicates from each group were analyzed by Biotree Biotech Co., Ltd. For untargeted metabolomics, samples were analyzed on a Vanquish UHPLC system interfaced with an Orbitrap Exploris 120 high-resolution mass spectrometer (Thermo Fisher Scientific). Metabolite separation was achieved using a Waters ACQUITY UPLC BEH Amide column (2.1 mm × 50 mm, 1.7 μm), with signal acquisition performed under positive and negative ionization conditions. Metabolites differing among groups were defined by VIP > 1 and *p* < 0.05. The identified metabolites were subsequently mapped to Kyoto Encyclopedia of Genes and Genomes pathways and analyzed for pathway enrichment (KEGG, RRID: SCR_012773). The resulting metabolomics data are accessible through the OMIX repository under accession OMIX016295, with BioProject PRJCA062324 (Jiang et al., 2026b).

### 2.10 *In vivo* SAM intervention

To validate the functional role of S-adenosylmethionine (SAM) in vivo, mice were randomly allocated to six groups: NC, SD, SD + SAM, SD + SAM + MLA, SAM, and MLA. SAM (Zeye Biotechnology, Cat# ZY1CA1252) and methyllycaconitine (MLA; MedChemExpress, Cat# HY-N2332A, purity 98.88%), an α7nAChR antagonist, were prepared in sterile PBS before use. The in vivo doses of SAM (100 mg/kg) and MLA (6 mg/kg) were selected based on published studies using SAM in cognitive or neuroprotective models and MLA for α7nAChR blockade (Kita et al., 2014; Wazea et al., 2018; Beauchamp et al., 2020; Zhang et al., 2023). All interventions were administered daily for 7 days. In the SD-related groups, drug administration was performed in parallel with the sleep deprivation procedure. To minimize handling-related bias, the routes of vehicle delivery were balanced among the corresponding experimental groups. Mice in the SAM and SD + SAM groups received SAM by oral gavage at 100 mg/kg. Mice assigned to the MLA group were treated with MLA intraperitoneally at 6 mg/kg. In the SD + SAM + MLA group, MLA was injected intraperitoneally at 6 mg/kg, followed 30 min later by oral administration of SAM at 100 mg/kg. Behavioral tests and hippocampal biochemical analyses were conducted after the intervention period.

### 2.11 BV2/HT22 non-contact coculture and *in vitro* treatments

Cell viability following S-adenosylmethionine (SAM), methyllycaconitine (MLA), or lipopolysaccharide (LPS from *Escherichia coli* O55:B5; Yeasen Biotechnology, Cat# 60747ES) exposure was assessed with a Cell Counting Kit-8 (Dojindo, Cat# CK04) assay to define non-cytotoxic working concentrations. Accordingly, the in vitro concentrations of SAM (400 μM) and MLA (20 μM) were chosen according to the CCK-8 screening results and the cell-based concentration ranges reported by Zheng et al. (2014) and Zhao et al. (2017). The full assay protocol is described in Supplementary Methods 1.2. For the non-contact BV2/HT22 coculture model, 24-well Transwell inserts with 0.4-μm pores (Corning, Cat# 3422) were employed. BV2 microglia were plated in the upper inserts at 5 × 10^4^ cells/well, while HT22 hippocampal neuronal cells were added to the lower wells at 1 × 10^5^ cells/well. Cocultures were maintained in 5% fetal bovine serum-containing medium.

After 24 h of coculture, cells were divided into six experimental groups: control, LPS, SAM, MLA, LPS + SAM, and LPS + SAM + MLA. Cells in the control group received no drug treatment. Cells in the LPS group were stimulated with LPS at 1 μg/mL for 24 h. Cells in the SAM group were treated with SAM at 400 μM, and cells in the MLA group were treated with MLA at 20 μM under the same timing scheme used for the corresponding pretreatment groups. In the LPS + SAM group, cells were pretreated with SAM at 400 μM for 30 min, followed by LPS stimulation at 1 μg/mL for 24 h. In the LPS + SAM + MLA group, cells were first pretreated with MLA at 20 μM for 30 min, then treated with SAM at 400 μM for 30 min, and finally stimulated with LPS at 1 μg/mL for 24 h. After treatment, cell pellets were collected for subsequent Western blot analysis.

### 2.12 Western blot analysis of signaling pathways

Total protein was prepared from cultured cells and hippocampal samples in RIPA buffer (Beyotime, Cat# P0013B), followed by concentration determination with the BCA method (Beyotime, Cat# P0012). The normalized lysates were loaded in equal amounts, resolved by SDS-PAGE and transferred to PVDF membranes (Millipore, Cat# IPVH00010). After nonspecific binding was blocked with 5% non-fat milk (Yam Bio, Cat# YM103) in TBST (Yam Bio, Cat# YM200), the blots were probed overnight at 4 °C with the indicated primary antibodies, followed by incubation with HRP-conjugated goat anti-rabbit IgG (CWBIO, Cat# CW0103S, RRID: AB_3251479) or HRP-conjugated goat anti-mouse IgG (CWBIO, Cat# CW0102S, RRID: AB_2736997), as appropriate.

The membranes were incubated with primary antibodies against α7 nicotinic acetylcholine receptor (α7nAChR; Affinity Biosciences, Cat# DF13247, RRID: AB_2846266), phospho-JAK2 (Cell Signaling Technology, Cat# 3771, RRID: AB_330403), JAK2 (Cell Signaling Technology, Cat# 3230, RRID: AB_2128522), STAT3 (Cell Signaling Technology, Cat# 4904, RRID: AB_331269), phospho-STAT3 (Cell Signaling Technology, Cat# 9145, RRID: AB_2491009), NF-κB p65 (Cell Signaling Technology, Cat# 8242, RRID: AB_10859369), phospho-NF-κB p65 (Cell Signaling Technology, Cat# 3033, RRID: AB_331284), and *β*-actin (Proteintech, Cat# 66009-1-Ig, RRID: AB_2687938).

Band intensities were captured using a Tanon 5,200 imaging system and processed with ImageJ (RRID: SCR_003070). For quantification, total protein values were adjusted using *β*-actin as the internal reference, while phosphorylated proteins were represented as the phosphorylated-to-total protein ratio.

### 2.13 Statistical analysis

All quantitative results are shown as the mean ± standard error of the mean (SEM). Statistical analyses were conducted using GraphPad Prism 9.0 (RRID: SCR_002798). Student’s *t*-test was applied for two-group comparisons, whereas one-way analysis of variance (ANOVA) was used for comparisons involving more than two groups. Escape latency across the training days of the Morris water maze test was evaluated using two-way repeated-measures ANOVA. A *p* value < 0.05 was considered statistically significant.

## 3 Results

### 3.1 *Lactobacillus acidophilus* improves sleep deprivation-associated microbial disruption and colonic barrier damage

Initial 16S rRNA gene sequencing showed that SD reduced intestinal microbial diversity and altered the overall community structure, as visualized by PCA and hierarchical clustering analyses. In addition, SD decreased the abundance of taxa belonging to the genus Lactobacillus in fecal samples (Supplementary Figure 1). Considering the reported cognitive-related effects and translational probiotic relevance of *L. acidophilus*, *L. acidophilus* CICC 22162 was selected as a representative exogenous probiotic intervention strain to evaluate whether Lactobacillus-based supplementation could alleviate SD-associated gut microbial disturbance and cognitive impairment.

**Figure 1:**
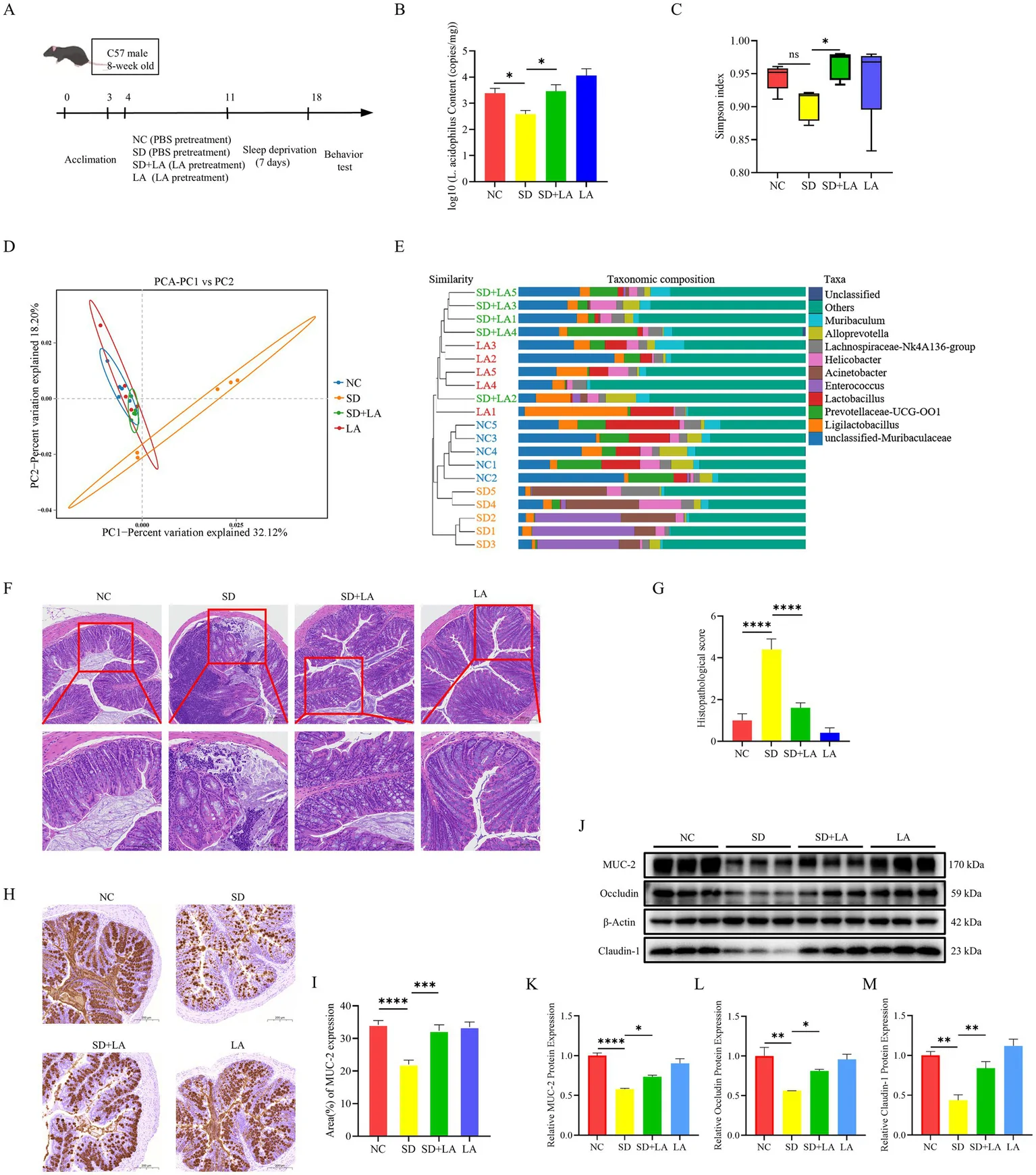
*Lactobacillus acidophilus* mitigates SD-associated gut microbial imbalance and colonic barrier disruption. **(A)** Schematic overview of the experimental design. **(B)** Absolute qPCR quantification of fecal *L. acidophilus* abundance across groups. **(C)** Gut microbial alpha diversity at the operational taxonomic unit (OTU) level, represented by the Simpson index. **(D)** Principal component analysis (PCA) of gut microbial profiles. **(E)** Hierarchical clustering of gut microbial communities. **(F)** Representative hematoxylin and eosin (H&E)-stained colon sections. **(G)** Histopathological scoring of colonic tissues. **(H)** Representative MUC2 immunohistochemical staining in colon sections. **(I)** Quantitative analysis of the MUC2-positive area. **(J)** Representative western blot bands for MUC2, Occludin, and Claudin-1 in colonic tissues. **(K–M)** Quantification of MUC2 **(K)**, Occludin **(L)**, and Claudin-1 **(M)** protein abundance. Protein abundance was normalized to *β*-actin and expressed relative to the NC group. Sample sizes were *n* = 5 per group for panels **(B–G)**, *n* = 6 per group for panel **(I)**, and *n* = 3 per group for panels **(K–M)**. Values are shown as mean ± SEM. Significance thresholds were defined as follows: ns, *p* ≥ 0.05; * *p* < 0.05; ** *p* < 0.01; *** *p* < 0.001; **** *p* < 0.0001. Scale bars: 100 μm and 200 μm in panel **(F)**; 200 μm in panel **(H)**.

To determine whether *L. acidophilus* modulated the intestinal microbial ecosystem and barrier status under SD conditions, mice were divided into NC, SD, SD + LA, and LA groups according to the experimental design shown in Figure 1A. Absolute quantitative PCR confirmed higher fecal *L. acidophilus* levels in the SD + LA and LA groups than in their corresponding saline-treated controls (Figure 1B). SD produced a marked shift in the gut microbial profile, whereas *L. acidophilus* administration partially counteracted this alteration. The Simpson index declined after SD exposure and increased following *L. acidophilus* treatment (Figure 1C). PCA placed NC and SD samples into distinct clusters, while samples from the SD + LA group moved closer to the NC cluster (Figure 1D). Hierarchical clustering produced a comparable grouping pattern (Figure 1E).

Colonic histology further indicated mucosal damage in SD mice, characterized by epithelial loss, disorganized crypt architecture, and inflammatory cell infiltration. These lesions were less pronounced after *L. acidophilus* administration (Figures 1F,G). MUC2 immunostaining also showed weaker mucus-associated staining after SD, whereas *L. acidophilus* treatment increased the MUC2-positive area (Figures 1H,I). Consistently, Western blotting showed lower Occludin, Claudin-1, and MUC2 protein abundance in SD mice, with partial recovery after *L. acidophilus* intervention (Figures 1J–M). Across most measured indices, the LA group remained comparable to the NC group. These results indicate that *L. acidophilus* helps restore microbial and barrier homeostasis disrupted by SD.

### 3.2 *Lactobacillus acidophilus* alleviates cognitive impairment and hippocampal injury in sleep-deprived mice

**Figure 2:**
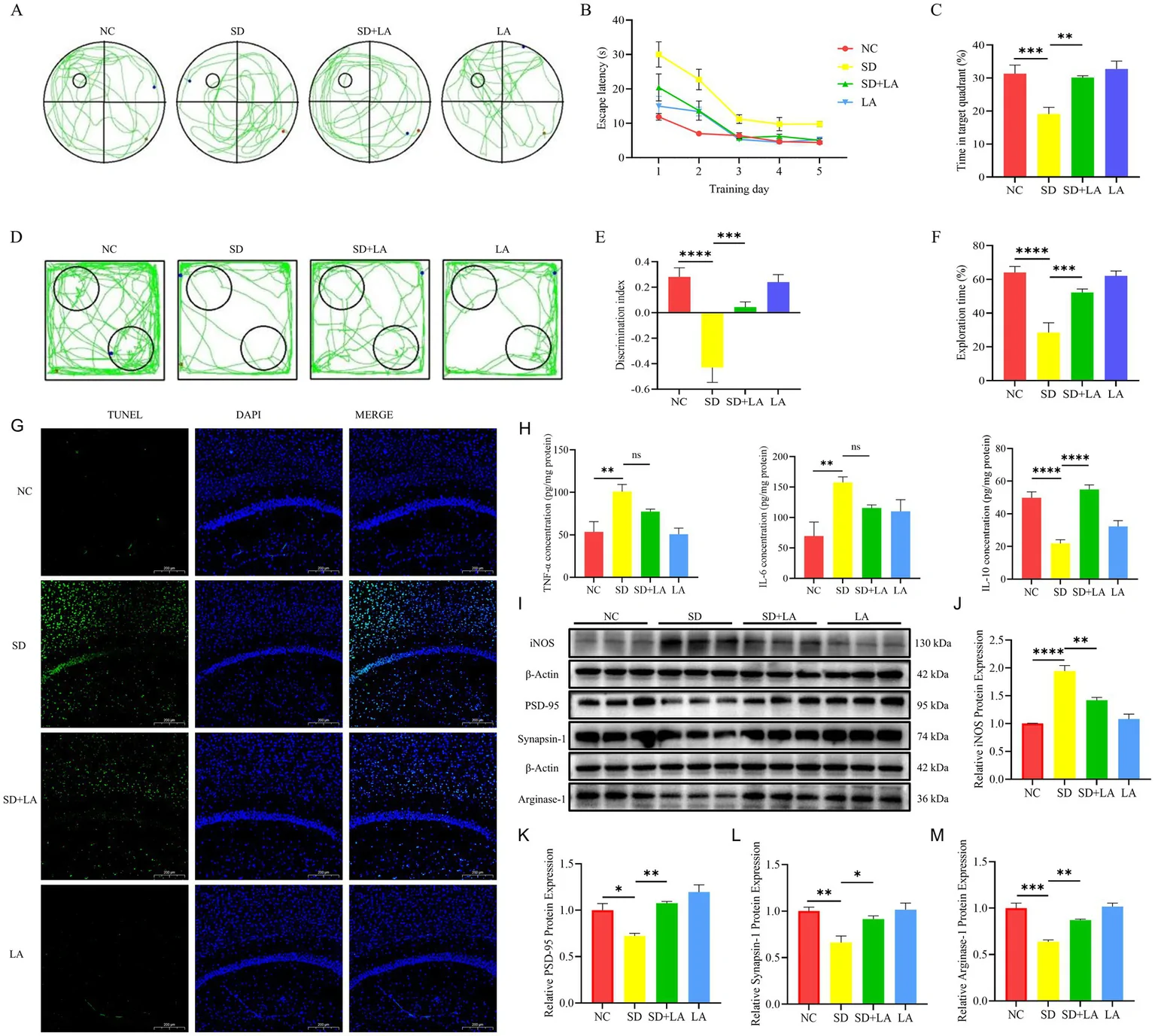
*Lactobacillus acidophilus* mitigates SD-associated cognitive deficits and hippocampal injury. **(A)** Representative swim paths in the Morris water maze. **(B)** Training-phase escape latency in the Morris water maze. **(C)** Target-quadrant residence during the probe trial. **(D)** Representative exploration traces in the novel object recognition assay. **(E)** Discrimination index in the novel object recognition assay. **(F)** Exploration time directed toward the novel object. **(G)** Representative TUNEL staining in hippocampal sections. **(H)** ELISA-based quantification of hippocampal TNF-*α*, IL-6, and IL-10 levels. **(I)** Representative Western blot bands for iNOS, PSD-95, Synapsin-1, and Arginase-1 in hippocampal tissues. **(J–M)** Quantification of iNOS **(J)**, PSD-95 **(K)**, Synapsin-1 **(L)**, and Arginase-1 **(M)** protein expression. Additional behavioral and inflammatory indices are provided in Supplementary Figure 2. Sample sizes were *n* = 7 per group for behavioral panels **(A–F)**, *n* = 3 per group for TUNEL staining in panel **(G)**, *n* = 6 per group for ELISA measurements in panel **(H)**, and *n* = 3 per group for Western blot analyses in panels **(I–M)**. Values are shown as mean ± SEM. Escape latency during training was analyzed using two-way repeated-measures ANOVA. For other quantified panels, significance thresholds were defined as follows: ns, *p* ≥ 0.05; * *p* < 0.05; ** *p* < 0.01; *** *p* < 0.001; **** *p* < 0.0001. Scale bar: 200 μm in panel **(G)**.

We next examined whether *L. acidophilus* alleviated SD-induced cognitive deficits and hippocampal injury. Representative trajectories from the Morris water maze are presented in Figure 2A. Relative to NC mice, SD-exposed mice required more time to locate the hidden platform during acquisition training and spent less time in the target quadrant in the probe test (Figures 2B,C), indicating deficits in spatial learning and memory retention. These impairments were attenuated by *L. acidophilus*, as shown by reduced platform-search latency and greater target-quadrant occupancy.

Representative exploration tracks from the novel object recognition test are shown in Figure 2D, reflecting recognition-memory performance across groups. SD mice displayed a lower discrimination index and reduced exploration of the novel object compared with NC mice (Figures 2E,F), whereas these behavioral deficits were partially reversed by *L. acidophilus* treatment. Additional behavioral tests showed that *L. acidophilus* also improved SD-associated abnormalities in Y-maze performance and reduced anxiety-like behavior in the elevated plus maze (Supplementary Figures 2A–F).

Hippocampal apoptosis and inflammatory markers were then examined. Consistent with tissue injury, SD increased the hippocampal TUNEL-positive signal, whereas *L. acidophilus* administration attenuated this apoptotic response (Figure 2G). ELISA analysis showed that SD shifted the hippocampal cytokine profile toward a pro-inflammatory state, with increased TNF-*α* and IL-6 levels and decreased IL-10 levels. This inflammatory imbalance was partly restored by *L. acidophilus* administration (Figure 2H). Additional ELISA results for Arginase-1, IL-1*β*, and IL-4 are presented in Supplementary Figure 2G.

Consistent with these behavioral and inflammatory changes, Western blot analysis showed that SD increased hippocampal iNOS expression and reduced the expression of Arginase-1, Synapsin-1, and PSD-95. These protein alterations were partially reversed after *L. acidophilus* treatment (Figures 2I–M). For most measured outcomes, the LA group remained comparable to the NC group. These findings indicate that *L. acidophilus* alleviates SD-related cognitive dysfunction and is associated with reduced hippocampal inflammation, neuronal apoptosis, and synaptic protein loss.

### 3.3 Fecal microbiota transfer indicates that gut microbiota participates in *Lactobacillus acidophilus*-associated cognitive benefits

**Figure 3:**
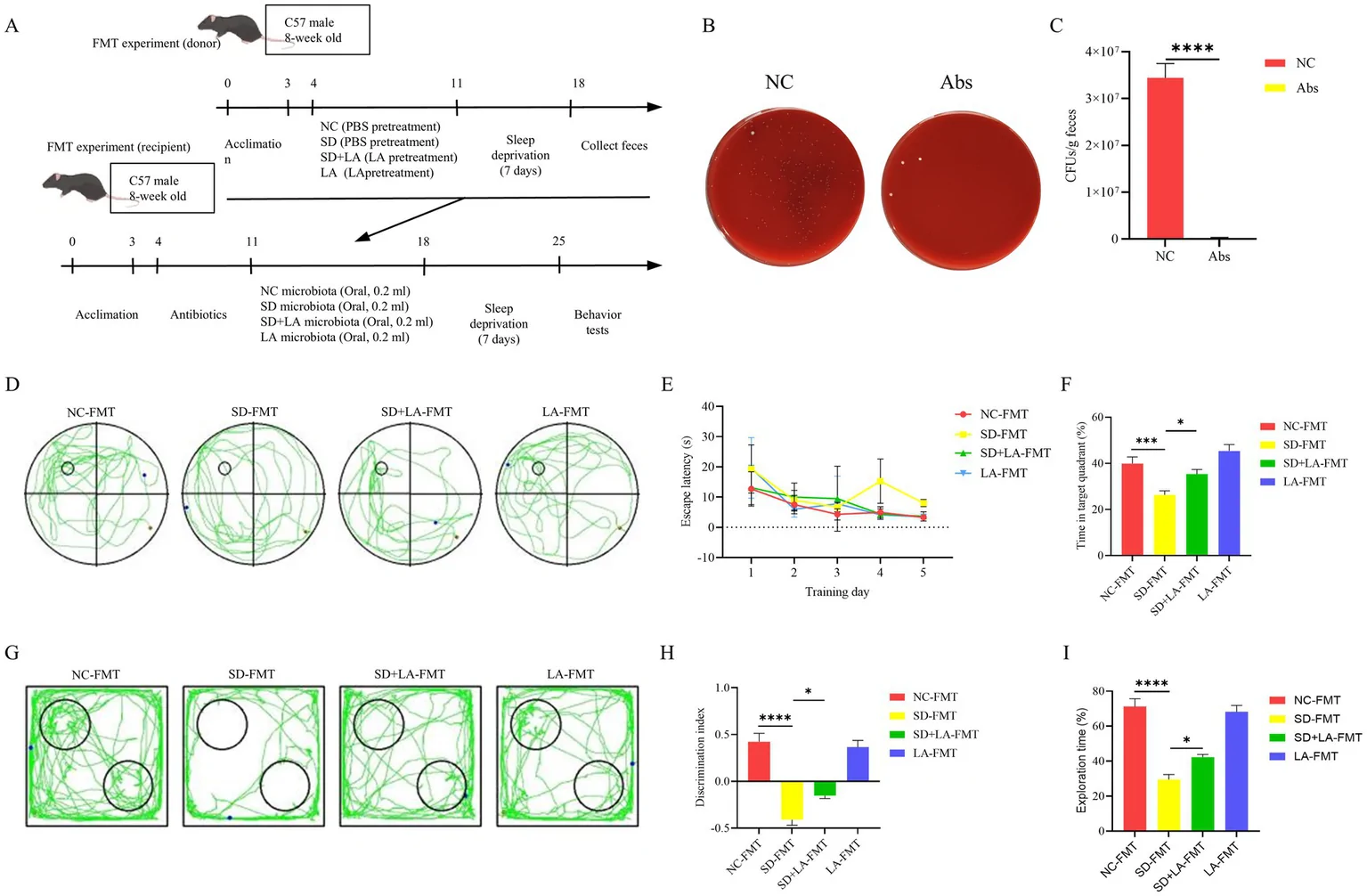
Microbiota transfer partially conveys the cognitive benefits associated with *L. acidophilus* treatment. **(A)** Workflow of the fecal microbiota transplantation (FMT) experiment. **(B)** Columbia blood agar culture used to verify depletion of resident gut microbiota in recipient mice before FMT. **(C)** Quantification of colony numbers from panel **(B)**. **(D)** Representative swim paths in the Morris Water Maze. **(E)** Training-phase escape latency in the Morris Water Maze. **(F)** Target-quadrant residence during the probe trial. **(G)** Representative exploration traces in the Novel Object Recognition Assay. **(H)** Discrimination index in the Novel Object Recognition Assay. **(I)** Exploration time directed toward the novel object. Additional behavioral outcomes and hippocampal injury-related indices in recipient mice are provided in Supplementary Figure 3, and intestinal barrier-related results are provided in Supplementary Figure 4. Sample sizes were *n* = 5 per group for panels **(B,C)** and *n* = 7 per group for behavioral panels **(D–I)**. Values are shown as mean ± SEM. Escape latency during training was analyzed using two-way repeated-measures ANOVA. For the remaining quantified panels, significance thresholds were defined as follows: ns, *p* ≥ 0.05; * *p* < 0.05; ** *p* < 0.01; *** *p* < 0.001; **** *p* < 0.0001.

We next examined whether the gut microbial community functionally contributes to the protective effects associated with *L. acidophilus*. To address this, fecal microbiota transplantation (FMT) was conducted according to the workflow illustrated in Figure 3A. Before FMT, antibiotic pretreatment markedly reduced culturable fecal bacteria in recipient mice, as assessed by colony counts on Columbia blood agar plates (Figures 3B,C).

Recipient mice received fecal suspensions prepared from NC, SD, SD + LA, or LA donor mice and were subsequently exposed to sleep deprivation for 7 days. Representative swim paths are shown in Figure 3D. In the Morris water maze, mice receiving microbiota from SD donors (SD-FMT) required more time to locate the hidden platform during training and showed reduced target-quadrant residence during the probe trial compared with NC-FMT mice (Figures 3E,F), indicating impaired spatial memory performance. By contrast, recipient mice given microbiota from SD + LA donors performed better than SD-FMT mice in these Morris water maze indices.

Recognition memory followed a similar pattern. Representative exploration traces are presented in Figure 3G. Compared with NC-FMT mice, SD-FMT mice displayed a lower discrimination index and reduced exploration of the novel object (Figures 3H,I). These behavioral deficits were partially reversed in mice receiving microbiota from SD + LA donors.

Additional behavioral assays further showed that microbiota from SD + LA or LA donors improved SD-associated abnormalities in spatial working memory and anxiety-like behavior in recipient mice (Supplementary Figures 3A–F). Consistent with these behavioral results, hippocampal apoptosis, inflammatory imbalance, synaptic protein disruption, and intestinal barrier injury were also reduced after transfer of microbiota from *L. acidophilus*-treated donors (Supplementary Figures 3G–M, 4A–H). These data indicate that the protective phenotype associated with *L. acidophilus* can be partially transmitted through the gut microbial community.

### 3.4 Untargeted metabolomics identifies S-adenosylmethionine as a candidate microbiota-associated metabolite after *Lactobacillus acidophilus* treatment

**Figure 4:**
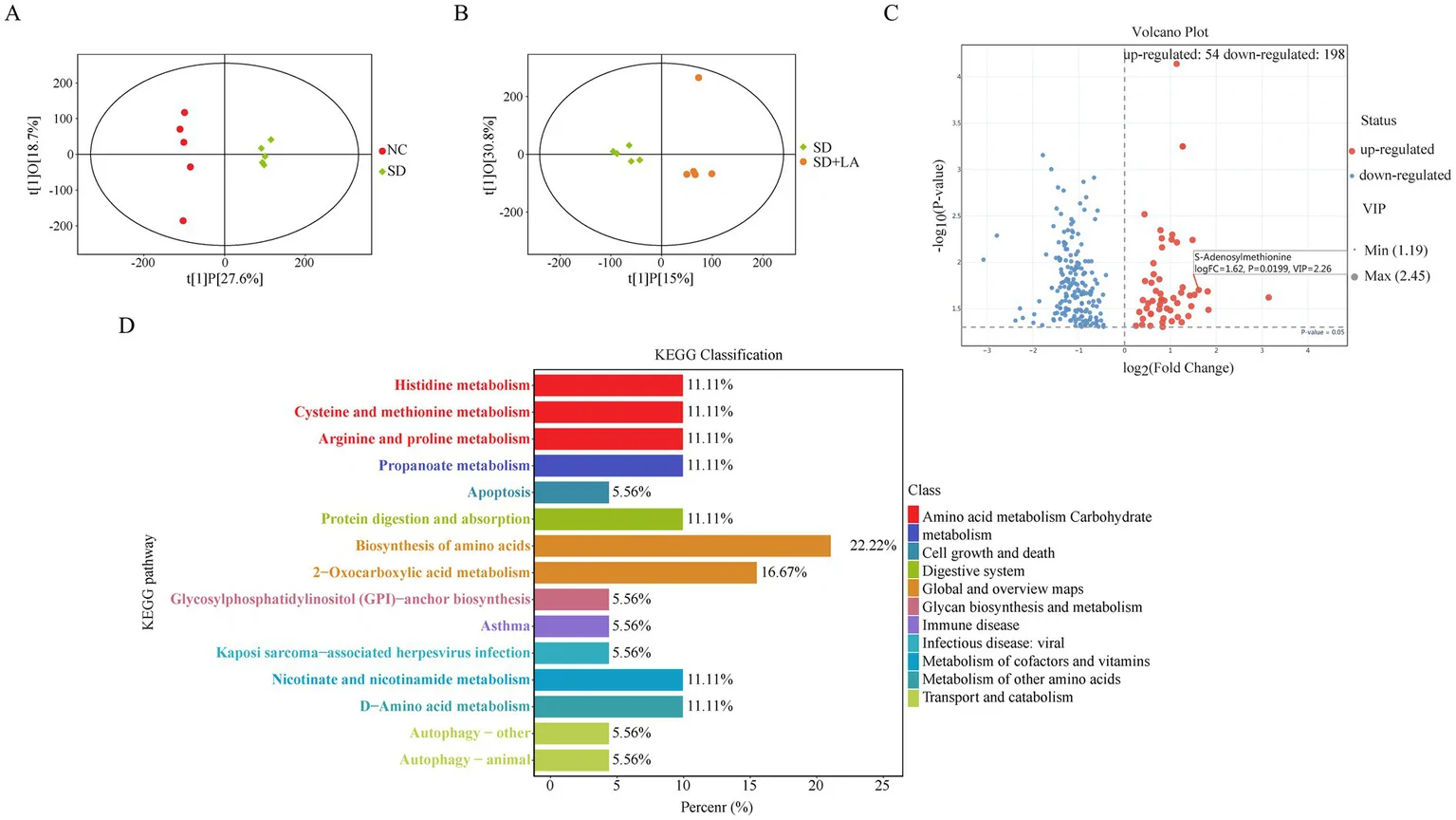
Fecal metabolomic profiling highlights S-adenosylmethionine after *L. acidophilus* administration. **(A)** Principal coordinate analysis (PCoA) comparing fecal metabolite patterns between the NC and SD groups. **(B)** PCoA comparing fecal metabolite patterns between the SD and SD + LA groups. **(C)** Volcano plot of metabolites altered in the SD versus SD + LA comparison. **(D)** Kyoto Encyclopedia of Genes and Genomes (KEGG) pathway mapping and enrichment analysis of altered metabolites from the SD versus SD + LA comparison. For metabolomic profiling, *n* = 5 per group.

Untargeted metabolomic profiling was performed to identify fecal metabolic changes associated with *L. acidophilus* intervention. Principal coordinates analysis (PCoA) showed distinct separation between the NC and SD groups and between the SD and SD + LA groups, suggesting that SD reshaped the fecal metabolite profile and that *L. acidophilus* treatment partly redirected this metabolic alteration (Figures 4A,B). Volcano plot analysis identified multiple metabolites with differential abundance between the SD and SD + LA groups. Among these metabolites, S-adenosylmethionine (SAM) was increased after *L. acidophilus* treatment (Figure 4C). Specifically, SAM was significantly increased in the SD + LA group compared with the SD group (VIP = 2.26, *p* = 0.0199, fold change = 3.07). Kyoto Encyclopedia of Genes and Genomes (KEGG) pathway enrichment analysis indicated that the altered metabolites were mainly associated with amino acid metabolism, with cysteine and methionine metabolism showing prominent enrichment (Figure 4D). Given that SAM was increased after *L. acidophilus* treatment, mapped to the enriched cysteine and methionine metabolism pathway, and has recognized biological relevance to methyl-donor metabolism and neuroimmune regulation, SAM was selected for further functional validation.

### 3.5 S-adenosylmethionine partially recapitulates the protective effects of *Lactobacillus acidophilus* in sleep-deprived mice

**Figure 5:**
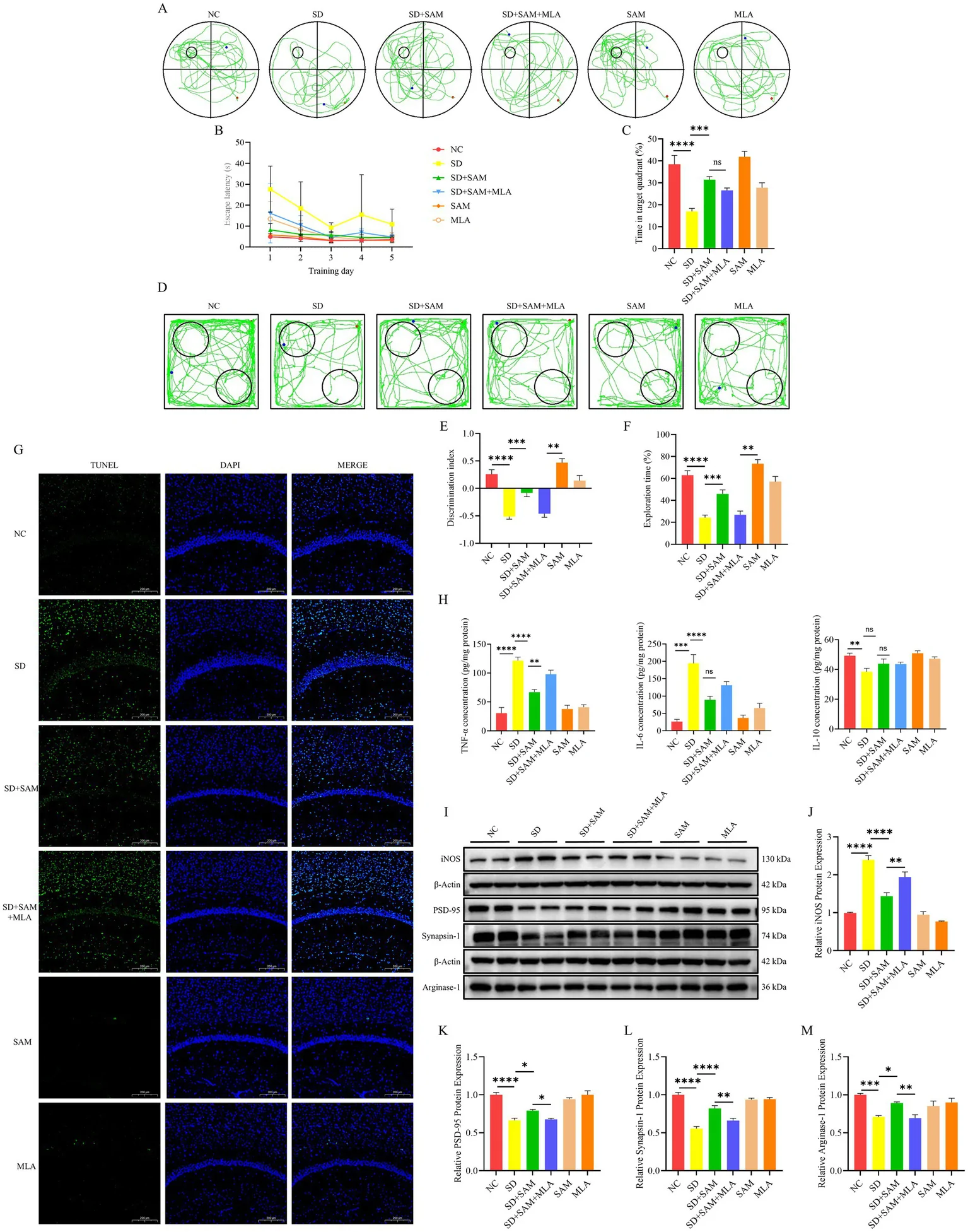
S-adenosylmethionine mitigates SD-associated cognitive deficits and hippocampal injury, whereas methyllycaconitine weakens this response. **(A)** Representative swim paths in the Morris water maze. **(B)** Training-phase escape latency in the Morris water maze. **(C)** Target-quadrant residence during the probe trial. **(D)** Representative exploration traces in the novel object recognition assay. **(E)** Discrimination index in the novel object recognition assay. **(F)** Exploration time directed toward the novel object. **(G)** Representative TUNEL staining in hippocampal sections. **(H)** ELISA-based quantification of hippocampal TNF-α, IL-6, and IL-10 levels. **(I)** Representative Western blot bands for iNOS, PSD-95, Synapsin-1, and Arginase-1 in hippocampal tissues. **(J–M)** Quantification of iNOS **(J)**, PSD-95 **(K)**, Synapsin-1 **(L)**, and Arginase-1 **(M)** protein expression. Additional behavioral outcomes are provided in Supplementary Figures 5A–F, and additional inflammatory markers are provided in Supplementary Figure 5G. Sample sizes were *n* = 6 per group for Morris water maze panels **(A–C)**, *n* = 7 per group for novel object recognition panels **(D–F)**, *n* = 3 per group for TUNEL staining in panel **(G)**, *n* = 6 per group for ELISA measurements in panel **(H)**, and *n* = 4 per group for Western blot analyses in panels **(I–M)**. Values are shown as mean ± SEM. Escape latency during training was analyzed using two-way repeated-measures ANOVA. For the remaining quantified panels, significance thresholds were defined as follows: ns, *p* ≥ 0.05; * *p* < 0.05; ** *p* < 0.01; *** *p* < 0.001; **** *p* < 0.0001.

To test whether S-adenosylmethionine (SAM) contributes to the protective effects observed with *L. acidophilus*, SD-exposed mice were treated with SAM alone or in combination with methyllycaconitine (MLA), a selective *α*7nAChR antagonist. In the Morris water maze, SAM improved spatial memory in SD mice, as shown by faster acquisition of the hidden-platform task and greater occupancy of the target quadrant in the probe trial (Figures 5A–C). In the novel object recognition test, SAM also increased the discrimination index and enhanced novel-object exploration in SD animals (Figures 5D–F). These behavioral improvements were weakened by co-administration of MLA.

A similar pattern emerged from additional behavioral assessments. SAM corrected SD-induced deficits in spatial working memory and reduced anxiety-like behavior, whereas MLA partially counteracted these improvements (Supplementary Figures 5A–F). TUNEL staining revealed that SAM reduced the SD-associated increase in hippocampal apoptotic cells, an effect that was diminished by MLA (Figure 5G). ELISA measurements further showed that SAM shifted the hippocampal cytokine balance away from a pro-inflammatory profile, decreasing TNF-α and IL-6 while raising IL-10. MLA attenuated this regulatory action (Figure 5H). Additional ELISA data for IL-1*β*, IL-4 and Arginase-1 are presented in Supplementary Figure 5G.

In agreement with these findings, immunoblotting demonstrated that SAM lowered iNOS expression and elevated Arginase-1, Synapsin-1 and PSD-95 in the hippocampus of SD mice. MLA co-therapy partially reversed these protein alterations (Figures 5I–M). Collectively, these results indicate that SAM recapitulates a portion of the *L. acidophilus*-associated protective phenotype, and point to the involvement of α7nAChR-related signaling in the regulation of SD-induced cognitive deficits and hippocampal damage.

### 3.6 S-adenosylmethionine attenuates microglial inflammatory activation through α7nAChR-associated regulation of JAK2/STAT3 and NF-κB signaling

To clarify how S-adenosylmethionine (SAM) modulates neuroinflammatory responses, a non-contact BV2/HT22 coculture system was used. Non-cytotoxic working concentrations of LPS, SAM, and methyllycaconitine (MLA), an α7nAChR antagonist, were first defined by CCK-8 assays (Supplementary Figure 6). BV2 microglia were then challenged with LPS under different treatment conditions, with or without SAM and MLA.

**Figure 6:**
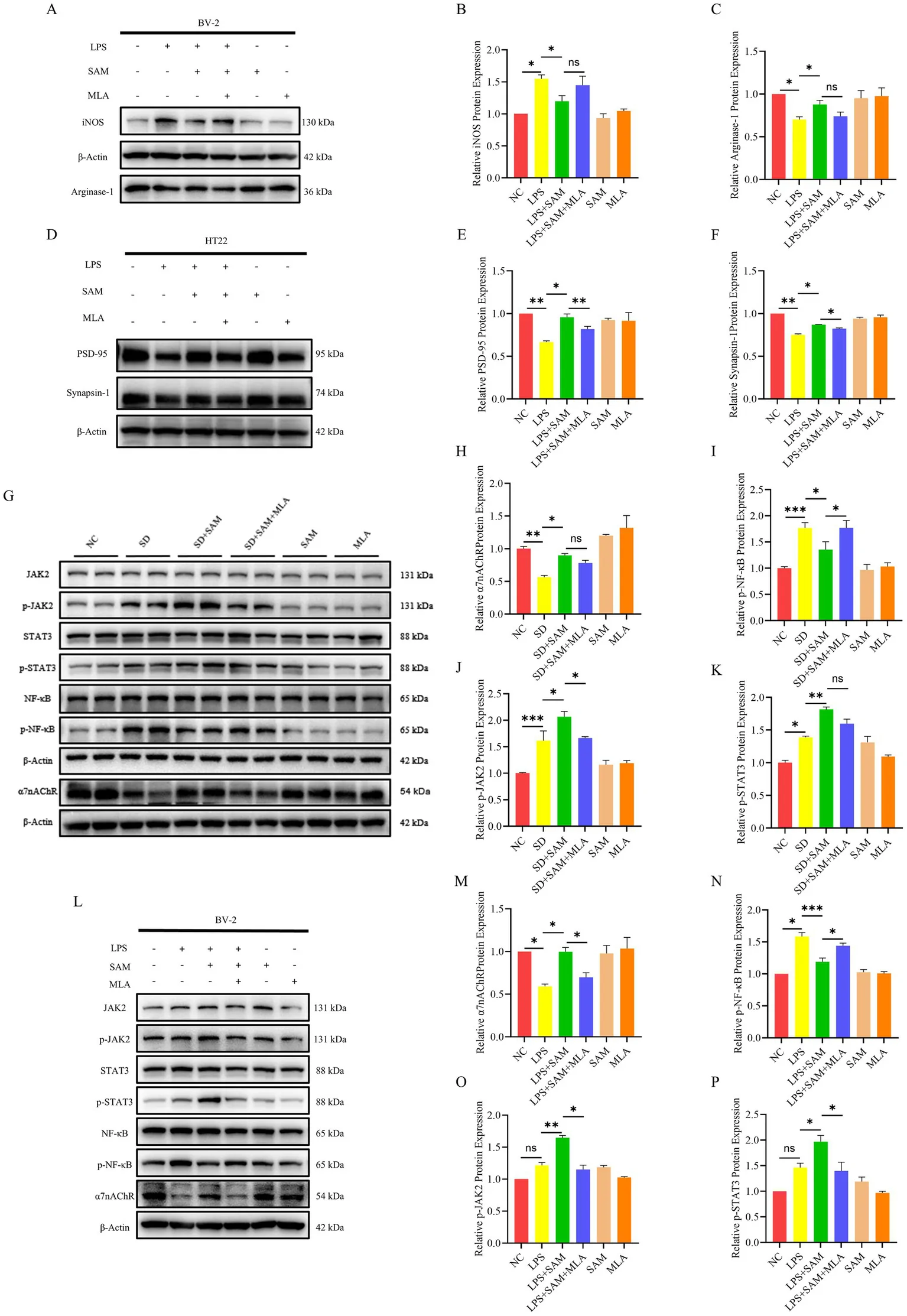
S-adenosylmethionine modulates microglial inflammatory activation, neuronal synaptic protein expression, and α7nAChR-related JAK2/STAT3 and NF-κB pathways. **(A)** Representative immunoblots for iNOS and Arginase-1 in BV2 cells. **(B,C)** Quantification of iNOS **(B)** and Arginase-1 **(C)** protein expression from panel **(A)**. **(D)** Representative immunoblots for PSD-95 and Synapsin-1 in HT22 cells. **(E,F)** Quantification of PSD-95 **(E)** and Synapsin-1 **(F)** protein expression from panel **(D)**. **(G)** Representative immunoblots for JAK2, p-JAK2, STAT3, p-STAT3, NF-κB p65, p-NF-κB p65, and α7nAChR in hippocampal tissues. **(H–K)** Quantification of α7nAChR **(H)**, p-NF-κB p65/NF-κB p65 **(I)**, p-JAK2/JAK2 **(J)**, and p-STAT3/STAT3 **(K)** in hippocampal tissues. **(L)** Representative immunoblots for JAK2, p-JAK2, STAT3, p-STAT3, NF-κB p65, p-NF-κB p65, and α7nAChR in BV2 cells from the BV2/HT22 non-contact coculture model. **(M–P)** Quantification of α7nAChR **(M)**, p-NF-κB p65/NF-κB p65 **(N)**, p-JAK2/JAK2 **(O)**, and p-STAT3/STAT3 **(P)** in BV2 cells. Total-protein signals were normalized to *β*-actin, whereas phosphorylated protein signals were calculated relative to their corresponding total protein forms. CCK-8 results used to define the working concentrations of LPS, SAM, and MLA are provided in Supplementary Figure 6. For *in vitro* Western blot analyses in panels **(A–F)** and **(L–P)**, *n* = 3 independent experiments per group. For hippocampal Western blot analyses in panels **(G–K)**, *n* = 4 mice per group. Values are shown as mean ± SEM. Significance thresholds were defined as follows: ns, *p* ≥ 0.05; * *p* < 0.05; ** *p* < 0.01; *** *p* < 0.001; **** *p* < 0.0001.

Immunoblotting showed that LPS increased iNOS expression and reduced Arginase-1 expression in BV2 cells, indicating inflammatory activation of microglia (Figures 6A–C). In the paired HT22 neuronal cells, LPS-stimulated BV2 activation was accompanied by lower PSD-95 and Synapsin-1 expression (Figures 6D–F). SAM treatment partially reversed these inflammatory and synaptic protein changes, whereas MLA pretreatment weakened the effect of SAM.

We then analyzed α7nAChR-related signaling in hippocampal tissues and in the BV2/HT22 coculture system. The total abundance of NF-κB p65, JAK2, and STAT3 remained largely unchanged among groups. By contrast, sleep deprivation *in vivo* or LPS stimulation *in vitro* increased the p-NF-κB p65/NF-κB p65 ratio and produced modest increases in the p-JAK2/JAK2 and p-STAT3/STAT3 ratios. Relative to the SD or LPS groups, SAM treatment increased α7nAChR expression, further elevated the p-JAK2/JAK2 and p-STAT3/STAT3 ratios, and lowered the p-NF-κB p65/NF-κB p65 ratio in hippocampal tissues (Figures 6G–K) and in BV2 cells from the coculture model (Figures 6L–P). MLA pretreatment partially reversed these signaling changes.

These data indicate that SAM restrains microglial inflammatory activation while preserving neuronal synaptic protein expression. This response is associated with α7nAChR-related enhancement of JAK2/STAT3 phosphorylation and concurrent suppression of NF-κB activation.

## 4 Discussion

Cognitive decline is strongly linked to insufficient sleep (sleep deprivation, SD). In our experimental model, SD triggered a consistent set of pathological changes: disruption of the gut microbial community, compromised intestinal barrier integrity, impaired learning and memory, activation of hippocampal inflammation, apoptosis of neurons, and reduction of synaptic proteins. Administration of *L. acidophilus* by oral gavage attenuated all these alterations. This constellation of results suggests that SD-induced cognitive impairment does not arise solely from central nervous system dysfunction; rather, it is accompanied by concurrent disturbances in the intestine and the gut microbiota along the microbiota–gut–brain axis. These observations align with earlier reports that have associated sleep loss with systemic dysregulation, disrupted memory processing, and cognitive deterioration (Rasch and Born, 2013; Kaneshwaran et al., 2019; Yuan and Wang, 2025).

Our microbiota analyses additionally support the idea that *L. acidophilus* helps regulate the intestinal ecosystem of SD-exposed animals. SD induced an overall shift in community composition, with a marked increase in potentially pro-inflammatory genera such as *Acinetobacter* and *Helicobacter*, while some beneficial genera including *Lactobacillus* decreased. Treatment with *L. acidophilus* partially counteracted these shifts. Quantitative PCR further confirmed that fecal *L. acidophilus* abundance increased after oral gavage. Of note, administration of *L. acidophilus* alone to normal mice did not appreciably change the gut microbiota, suggesting that the intervention did not disrupt microbial homeostasis under non-pathological conditions. These findings are consistent with earlier reports that SD can perturb gut microbial diversity and composition and promote dysbiosis-related inflammatory and cognitive abnormalities (Wang et al., 2021; Sun et al., 2023). More broadly, the microbiota–gut–brain axis has been recognized as an important route linking intestinal microbial changes with central nervous system injury, while probiotic or *L. acidophilus*-based interventions have been reported to influence inflammatory and cognitive outcomes (Huang Y. et al., 2025; Ma et al., 2025). Our findings also agree with previous observations that SD can induce gut dysbiosis, including alterations in *Lactobacillus*-related taxa (Wang et al., 2021; Sun et al., 2023).

The intestinal epithelium serves as a key boundary separating the gut lumen from host tissues. Prior work has demonstrated that SD-related gut microbial disruption can compromise epithelial barrier integrity and amplify inflammatory responses, thereby providing a route through which gut-derived mediators may affect peripheral tissues and the central nervous system (Wang et al., 2021; Li et al., 2023; Aburto and Cryan, 2024). In our experiments, *L. acidophilus* treatment improved colonic tissue morphology and raised the protein levels of MUC2, Occludin, and Claudin-1. This indicates that its protective effects go beyond simply altering the microbial community and include strengthening of the mucosal barrier, consistent with reports that *L. acidophilus* can enhance epithelial tight-junction function and restrain inflammatory signaling (Haque et al., 2024). Given that barrier breakdown can promote the spread of inflammatory signals from the gut to the brain, maintaining intestinal integrity may represent one pathway through which peripheral microbial regulation leads to reduced hippocampal damage (Dalile et al., 2019; Aburto and Cryan, 2024; O’Riordan et al., 2025).

The FMT results further indicate that the gut microbial community contributes functionally to the effects of *L. acidophilus*. In this experiment, recipient mice underwent microbiota depletion, received fecal microbiota from different donor groups, and were then subjected to SD. Therefore, the FMT data should be interpreted as evidence that donor microbiota modified the host response to a subsequent SD challenge. Recipients transplanted with microbiota from SD donors displayed poorer behavioral performance after SD, whereas microbiota from *L. acidophilus*-treated donors attenuated these abnormalities. Supplementary analyses showed parallel improvements in intestinal barrier status and hippocampal injury-related indices. These observations suggest that the protective phenotype may not depend solely on direct administration of the strain, but also on the microbial community structure and function shaped by *L. acidophilus* (Wang et al., 2021; Sun et al., 2023; O’Riordan et al., 2025). This interpretation is consistent with previous FMT studies showing that donor-derived gut microbial communities can transfer gut–brain-related behavioral and hippocampal phenotypes (De Palma et al., 2017; Rei et al., 2022). Therefore, microbiota alterations appear to participate actively in the pathological sequence linking SD to cognitive impairment, rather than merely accompanying sleep loss.

Another key result was the identification of S-adenosylmethionine (SAM) as a metabolite associated with *L. acidophilus*-mediated microbial remodeling and hippocampal protection. SAM was not selected solely based on untargeted metabolomics. Instead, it was selected based on its LA-associated increase in fecal metabolomic profiling, its involvement in the enriched cysteine and methionine metabolism pathway, its known biological relevance to methyl-donor metabolism and neuroimmune regulation, and its subsequent functional validation *in vivo* and *in vitro*. SAM intervention reproduced several *L. acidophilus*-associated effects, including improved cognitive performance, reduced neuronal apoptosis, attenuation of hippocampal inflammatory imbalance, and preservation of synaptic protein expression. These findings support SAM as a candidate microbiota-associated metabolic mediator linking peripheral microbial regulation to central neuroprotection. This interpretation supported by the work of Do Carmo et al., Zhang et al., and Zhao et al., who linked SAM can modulate cognitive performance, oxidative stress, neuroinflammation, and α7nAChR-related signaling in neurological disease models (Do Carmo et al., 2016; Zhang et al., 2023; Zhao et al., 2023). Nevertheless, because untargeted metabolomics is primarily a discovery-based strategy and detected multiple altered metabolites, SAM should be interpreted as a functionally validated candidate mediator rather than the only metabolite responsible for the protective effects of *L. acidophilus*. Additional microbial metabolites may also contribute to the protective effects of *L. acidophilus* (Dalile et al., 2019; Mukhopadhya and Louis, 2025; Wang et al., 2025).

The source of increased fecal SAM after *L. acidophilus* intervention also warrants further consideration. The present data do not distinguish whether *L. acidophilus* CICC 22162 directly contributes to SAM production or indirectly increases fecal SAM by remodeling microbial methionine/SAM metabolism. Wu et al. reported that gut microbiota participates in host methionine metabolism, which is closely associated with SAM biosynthesis (Wu et al., 2022). In addition, SAM-related biosynthetic capacity has been reported in lactic acid bacteria, as shown by the characterization of an SAM synthetase gene from *Lactobacillus paraplantarum* (Cha et al., 2015). More recently, a high-SAM-producing probiotic strain, *Lactobacillus helveticus* CCFM1320, was reported to alleviate sleep deprivation-induced cognitive and circadian abnormalities, supporting the biological plausibility of probiotic-associated SAM regulation (Tian et al., 2026). Therefore, the increase in fecal SAM observed in the present study may reflect direct strain-associated SAM metabolism, indirect regulation of gut microbial methionine/SAM metabolism, or host–microbiota metabolic interactions. Future studies using targeted LC–MS/MS or HPLC-based SAM quantification, in vitro fermentation of *L. acidophilus* CICC 22162, and microbial functional gene analysis will help clarify the precise origin of SAM.

Mechanistic analyses further connected SAM action with *α*7nAChR-related signaling. In mouse hippocampal tissues and the BV2/HT22 coculture system, SAM increased α7nAChR expression, reduced phosphorylated NF-κB p65, and enhanced JAK2 and STAT3 phosphorylation. These responses were weakened by the α7nAChR antagonist methyllycaconitine. This pattern is broadly consistent with previous work placing α7nAChR within cholinergic anti-inflammatory regulation, while NF-κB and JAK2/STAT3 represent downstream signaling modules that shape inflammatory tone and protective cellular responses (Wang et al., 2003; de Jonge et al., 2005; Egea et al., 2015). Notably, the overall abundance of NF-κB p65, JAK2, and STAT3 changed little across groups, whereas their phosphorylation status showed clearer variation. This finding suggests that SAM mainly regulates pathway activity rather than total protein expression.

When SD or LPS was applied alone, a moderate increase in p-JAK2 and p-STAT3 was observed. This response may reflect an early and incomplete stress-adaptive signal triggered by inflammatory stimulation, rather than a fully protective activation. This interpretation should remain cautious, because STAT3 activity can vary across biological contexts, including cellular background, stimulus strength, and disease progression. In our experimental model, SAM-driven JAK2/STAT3 phosphorylation occurred together with higher α7nAChR expression, reduced NF-κB activation, lower iNOS expression, a less pro-inflammatory cytokine profile marked by reduced TNF-α and IL-6, and increased IL-10 and Arginase-1. This response pattern is consistent with an anti-inflammatory and neuroprotective-like state under SAM treatment. The blunting of these changes by methyllycaconitine further suggests that α7nAChR participates, at least partly, in connecting SAM treatment with downstream JAK2/STAT3 and NF-κB regulation. Therefore, α7nAChR-related modulation of JAK2/STAT3 and NF-κB signaling may represent one mechanism through which SAM exerts its effects, although other pathways are also likely to be involved (Wang et al., 2003; Marrero and Bencherif, 2009).

Several caveats should be considered when interpreting these findings. First, the initial 16S rRNA gene sequencing data indicated changes in Lactobacillus-related taxa mainly at the genus level and did not resolve the specific endogenous Lactobacillus species involved. Given that mouse-adapted species such as *L. murinus* and *L. reuteri* are frequently detected in the murine intestine, *L. acidophilus* should be interpreted here as an exogenous probiotic intervention strain with reported cognitive-related relevance rather than as the key native Lactobacillus species driving the initial 16S signal (Peña et al., 2004; Jeon et al., 2022; Huang Y. et al., 2025). Second, the evidence was generated from mouse experiments and cell-based assays; therefore, whether these findings translate to human populations remains to be established. Considering the clinical burden of mild cognitive impairment and dementia (Hugo and Ganguli, 2014; Langa and Levine, 2014; Petersen et al., 2014), together with increasing interest in probiotic-based strategies for cognitive and sleep-related conditions (Patterson et al., 2024; Huang J. et al., 2025), future studies in clinical or population-based settings are needed. Third, although methyllycaconitine provided pharmacological evidence for α7nAChR involvement, more rigorous approaches, such as receptor-specific knockdown or knockout and complementary agonist-based experiments, would offer stronger mechanistic support. Finally, while untargeted metabolomics identified SAM as a candidate metabolite, targeted metabolomic validation and additional functional rescue experiments remain necessary. Future investigations should also address stress-related susceptibility to sleep loss (Kalmbach et al., 2018), astrocyte-associated neuroimmune mechanisms, and the broader neuropsychiatric consequences of gut dysbiosis.

Overall, this study indicates that *L. acidophilus* mitigates SD-related cognitive dysfunction through coordinated regulation of the gut microbiota, intestinal barrier integrity, microbiota-associated metabolism, and hippocampal inflammatory signaling. The gut microbiota–SAM–α7nAChR-related neuroimmune pathway may represent one mechanistic route through which *L. acidophilus* protects against sleep loss-associated cognitive impairment.

## Data Availability

The 16S rRNA amplicon sequencing data presented in this study can be found in the NCBI SRA repository (https://www.ncbi.nlm.nih.gov/sra) under accession number PRJNA1453587. The metabolome (mass spectrometry) data presented in this study can be found in the OMIX repository (https://ngdc.cncb.ac.cn/omix) under accession no. OMIX016295, associated with BioProject accession no. PRJCA062324.
