# Supplementary material for: Lactobacillus acidophilus CICC 22162 alleviates sleep deprivation-induced cognitive impairment by remodeling gut microbiota and enhancing S-adenosylmethionine-mediated neuroimmune regulation
Source: Front Microbiol. 2026 Aug 19;17:1889475. doi: 10.3389/fmicb.2026.1889475 (PMC13536946; doi:10.3389/fmicb.2026.1889475)
Supplement: Supplementary file 1 [file Data_Sheet_1.pdf]

Supplementary Material

1 Supplementary Methods

1.1 Cell culture

BV2 microglial cells and HT22 hippocampal neuronal cells were purchased from Procell Life Science & Technology Co., Ltd. Cells were cultured in DMEM supplemented with 10% fetal bovine serum and 1% penicillin–streptomycin in a humidified incubator at 37 °C with 5% CO2. Cells were passaged when they reached 80–90% confluence, and only cells in the logarithmic growth phase with good viability were used for subsequent experiments.

1.2 CCK-8 cell viability assay

Cell viability was assessed using a Cell Counting Kit-8 (CCK-8; Dojindo, Cat# CK04-11). BV2 and HT22 cells were seeded into 96-well plates at appropriate densities and allowed to attach overnight. After attachment, the cells were treated with different concentrations of S-adenosylmethionine (SAM; 0, 50, 100, 200, 400, and 800 μM; Zeye Biotechnology, Cat# ZY1CA1252), lipopolysaccharide (LPS; 0, 0.001, 0.01, 0.1, 1, and 10 μg/mL; Yeasen Biotechnology, Cat# 60747ES), or methyllycaconitine (MLA; 0, 2.5, 5, 10, and 20 μM; MedChemExpress, Cat# HY-N2332A). Each condition included six replicate wells. After 24 h of treatment, CCK-8 solution was added to each well according to the manufacturer's instructions, and the plates were incubated in the dark. Absorbance was then measured at 450 nm using a microplate reader. Cell viability was calculated relative to the untreated control and used to select non-cytotoxic concentrations for subsequent coculture experiments.

2 Supplementary Figures

2.1 Preliminary gut microbiota analysis in sleep-deprived mice

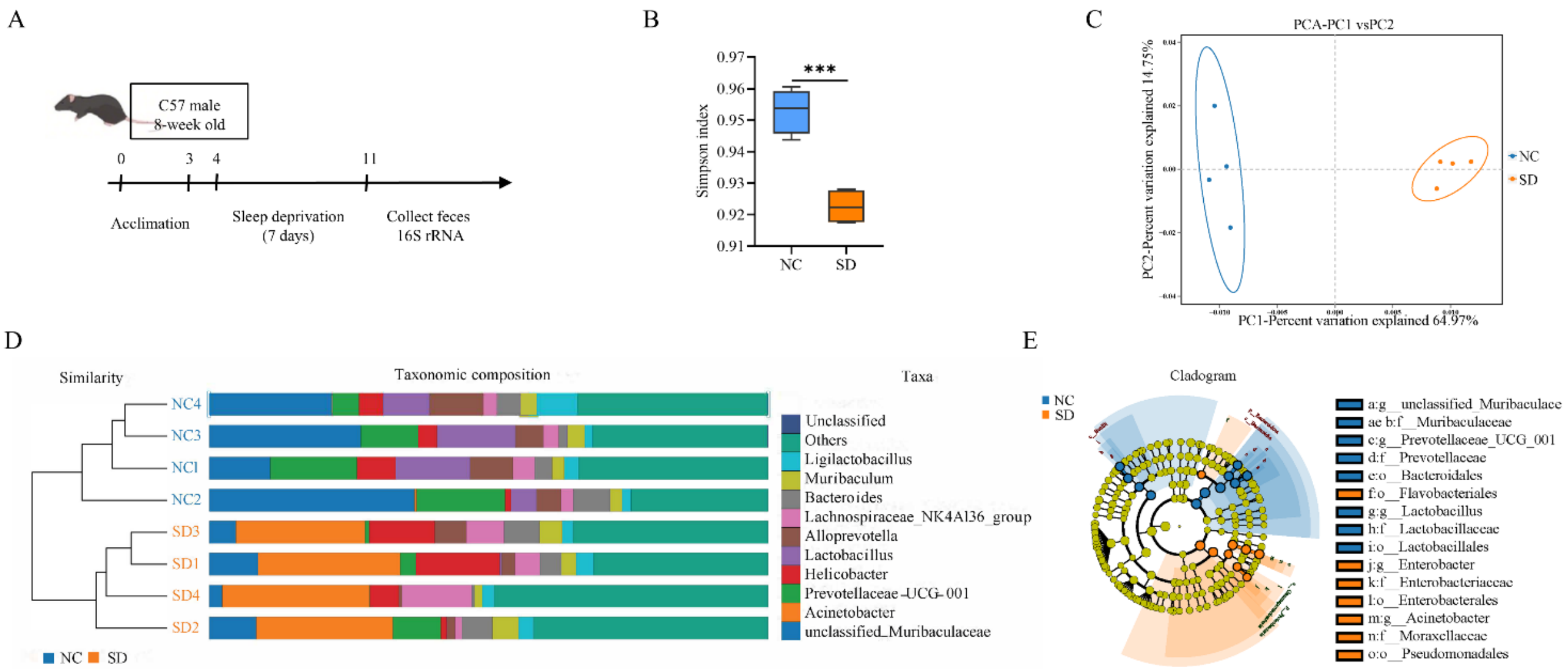

Supplementary Figure 1. Preliminary 16S rRNA gene sequencing analysis of gut microbiota in sleep-deprived mice. (A) Schematic diagram of the sleep deprivation model. (B) Alpha diversity of gut microbiota at the OTU level, shown as the Simpson index. (C) Principal component analysis (PCA) of gut microbiota composition. (D) Hierarchical clustering tree analysis of gut microbiota structure. (E) LEfSe cladogram analysis of differentially enriched taxa between the NC and SD groups. For gut microbiota analysis, n = 4 per group. For panel (B), data are presented as mean ± SEM. Statistical significance is indicated as ** $p < 0.01$.

2.2 Additional behavioral and inflammatory results in sleep-deprived mice

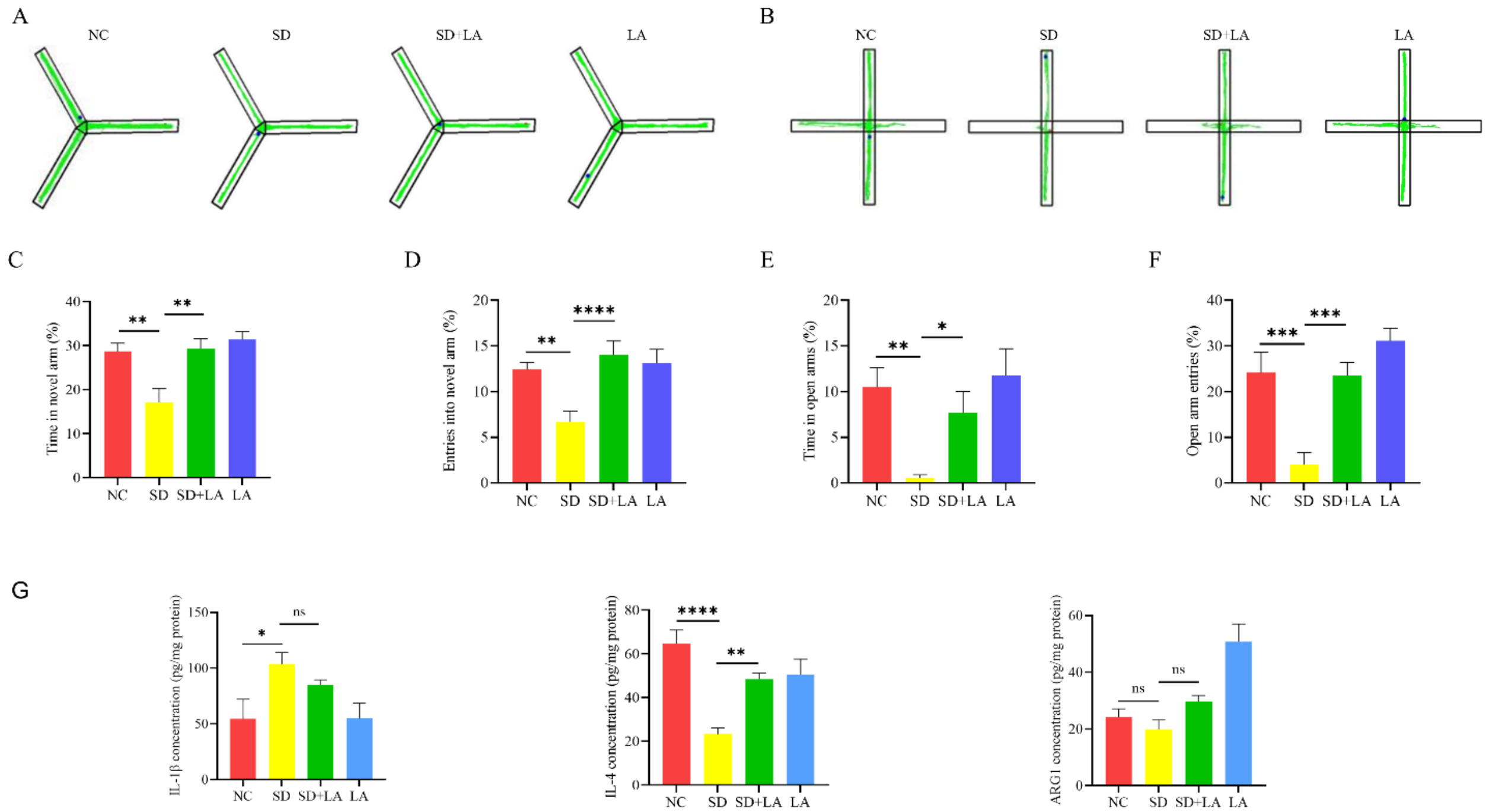

Supplementary Figure 2. Additional behavioral and inflammatory results in sleep-deprived mice.(A) Representative trajectories in the Y-maze test. (B) Percentage of time spent in the novel arm. (C) Percentage of entries into the novel arm. (D) Representative trajectories in the elevated plus maze test. (E) Percentage of time spent in the open arms. (F) Percentage of entries into the open arms. (G) ELISA analysis of Arginase-1, IL-1β, and IL-4 levels in hippocampal tissues. For behavioral tests in panels (A–F), n = 7 per group. For ELISA analysis in panel (G), n = 6 per group. Data are presented as mean ± SEM. Statistical significance is indicated as ns, $p \geq 0.05$; * $p < 0.05$; ** $p < 0.01$; *** $p < 0.001$; **** $p < 0.0001$.

2.3 Additional behavioral and hippocampal injury-related results in recipient mice after fecal microbiota transplantation

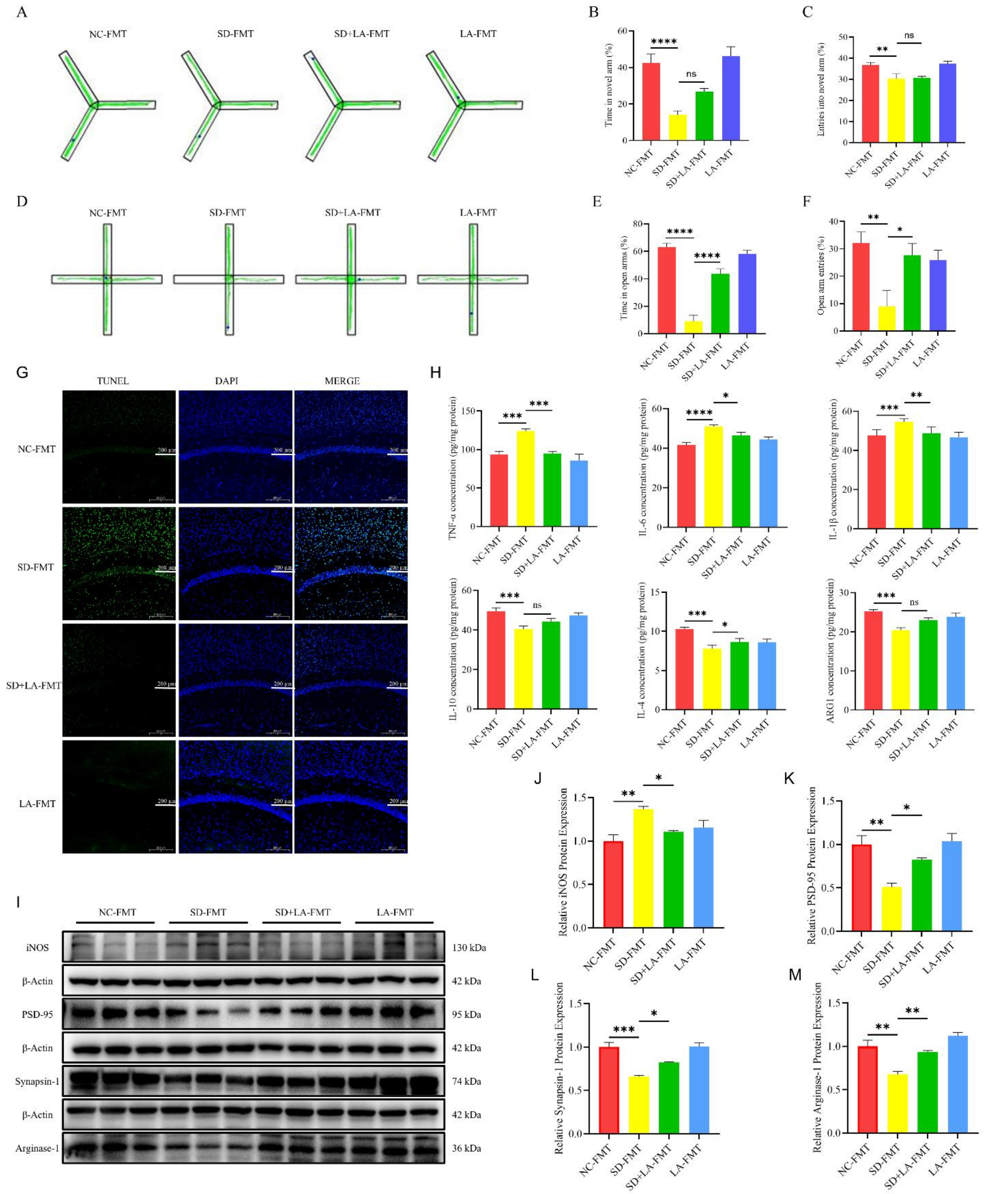

Supplementary Figure 3. Additional behavioral and hippocampal injury-related results in recipient mice after fecal microbiota transplantation. (A) Representative trajectories in the Y-maze test. (B) Percentage of time spent in the novel arm. (C) Percentage of entries into the novel arm. (D) Representative trajectories in the elevated plus maze test. (E) Percentage of time spent in the open

arms. (F) Percentage of entries into the open arms. (G) Representative TUNEL staining of hippocampal tissues in recipient mice. (H) ELISA analysis of IL-10, Arginase-1, IL-4, TNF-α, and IL-1β levels in hippocampal tissues of recipient mice. (I) Representative Western blot bands of iNOS, PSD-95, Synapsin-1, and Arginase-1 in hippocampal tissues of recipient mice. (J–M) Densitometric analysis of iNOS, PSD-95, Synapsin-1, and Arginase-1 protein expression, respectively. Protein levels were normalized to β-actin and expressed relative to the NC group. For Western blot analysis in panels (I–M), n = 3 per group. Data are presented as mean ± SEM. Statistical significance is indicated as ns, $p \geq 0.05$; * $p < 0.05$; ** $p < 0.01$; *** $p < 0.001$; **** $p < 0.0001$. Scale bar in (G) = 200 μm.

2.4 Lactobacillus acidophilus-associated donor microbiota alleviates intestinal barrier injury in recipient mice

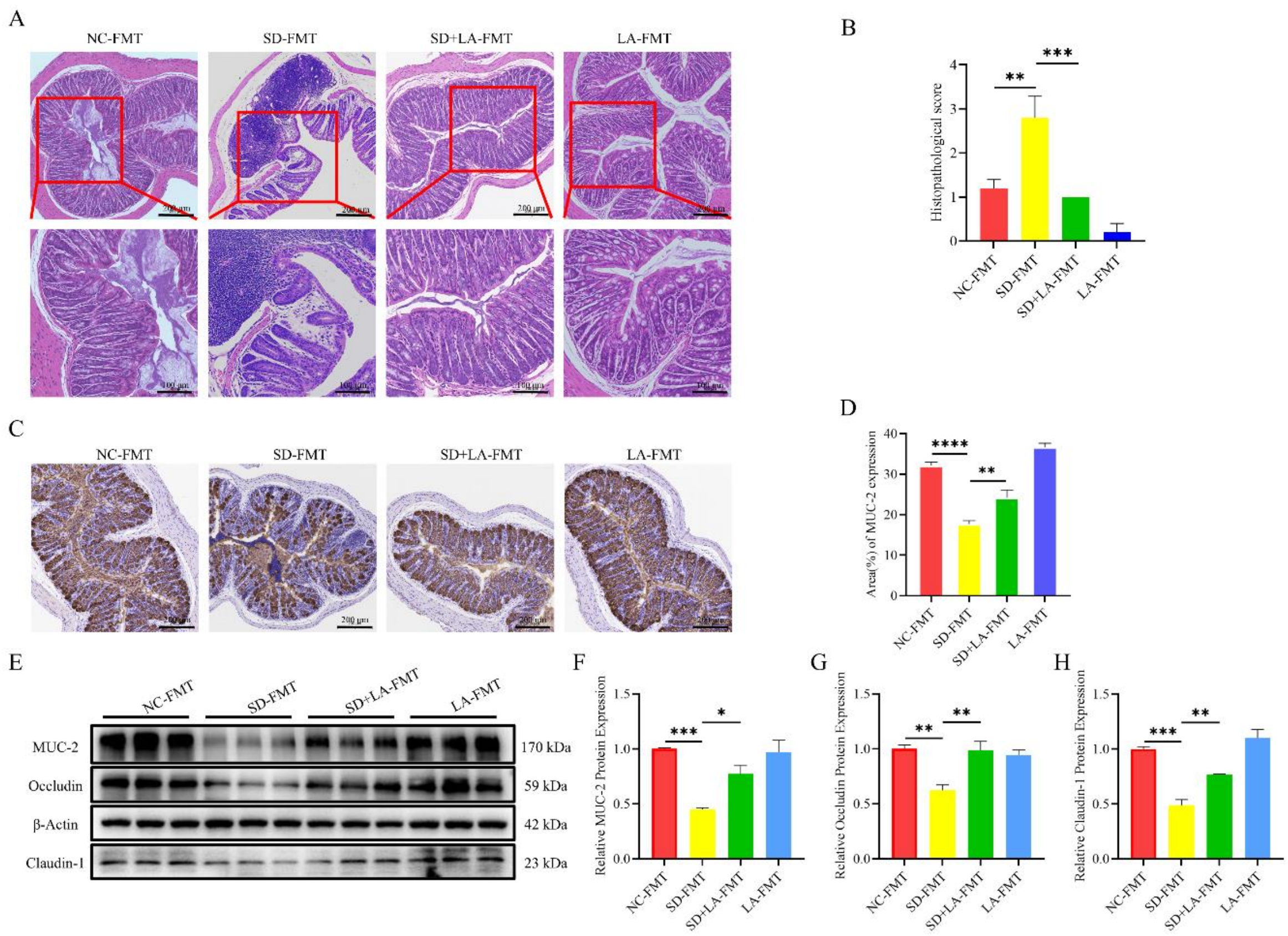

Supplementary Figure 4. Effects of Lactobacillus acidophilus-associated donor microbiota transplantation on colonic barrier-related parameters in recipient mice. (A) Representative H&E staining of colonic tissues in recipient mice. (B) Histopathological score analysis of colonic tissues in recipient mice. (C) Representative immunohistochemical staining of MUC2 in colonic tissues of recipient mice. (D) Quantification of the MUC2-positive area. (E) Representative Western blot bands of MUC2, Occludin, and Claudin-1 in colonic tissues of recipient mice. (F–H) Densitometric analysis of MUC2, Occludin, and Claudin-1 protein expression, respectively. Protein levels were normalized to β-actin and expressed relative to the NC group. For histopathological analysis in panels (A,B), n = 5 per group. For MUC2 immunohistochemical analysis in panels (C,D), n = 6 per group. For Western blot analysis in panels (E–H), n = 3 per group. Data are presented as mean ± SEM. Statistical

significance is indicated as ns, p ≥ 0.05; * p < 0.05; ** p < 0.01; *** p < 0.001; **** p < 0.0001. Scale bars: (A) 100 μm and 200 μm; (C) 200 μm.

2.5 Additional evidence that S-adenosylmethionine mimicked the neuroprotective effects of Lactobacillus acidophilus in sleep-deprived mice

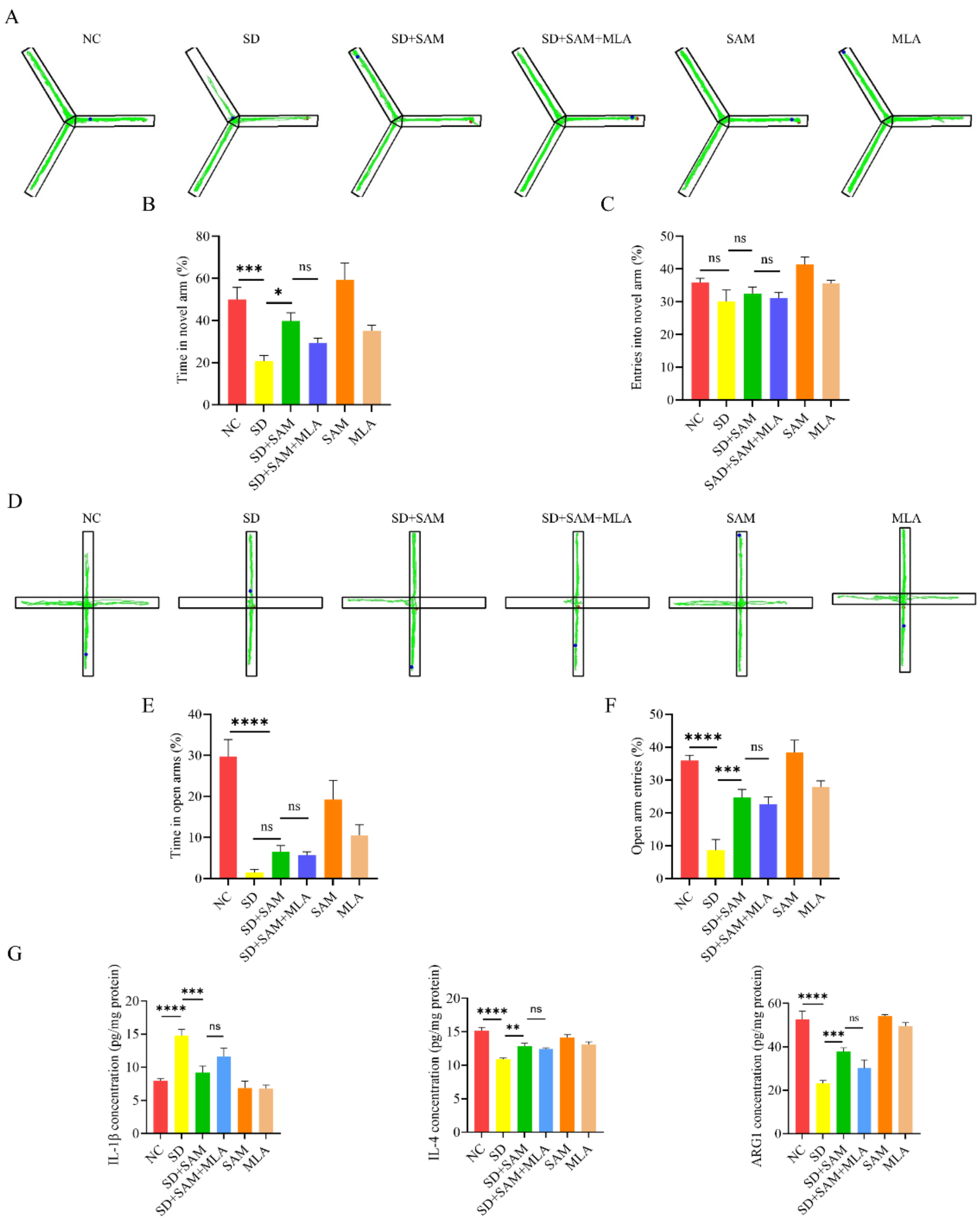

Supplementary Figure 5. Additional evidence that S-adenosylmethionine mimicked the neuroprotective effects of Lactobacillus acidophilus in sleep-deprived mice.(A) Representative trajectories in the Y-maze test. (B) Percentage of time spent in the novel arm. (C) Percentage of entries into the novel arm. (D) Representative trajectories in the elevated plus maze test. (E) Percentage of time spent in the open arms. (F) Percentage of entries into the open arms. (G) ELISA analysis of IL-1β, IL-4, and Arginase-1 levels in hippocampal tissues. For behavioral tests in panels

(A–F), n = 7 per group. For ELISA analysis in panel (G), n = 6 per group. Data are presented as mean ± SEM. Statistical significance is indicated as ns, $p \geq 0.05$; * $p < 0.05$; ** $p < 0.01$; *** $p < 0.001$; **** $p < 0.0001$.

2.6 CCK-8 assays for determining the working concentrations of LPS, SAM, and MLA in BV2 and HT22 cells

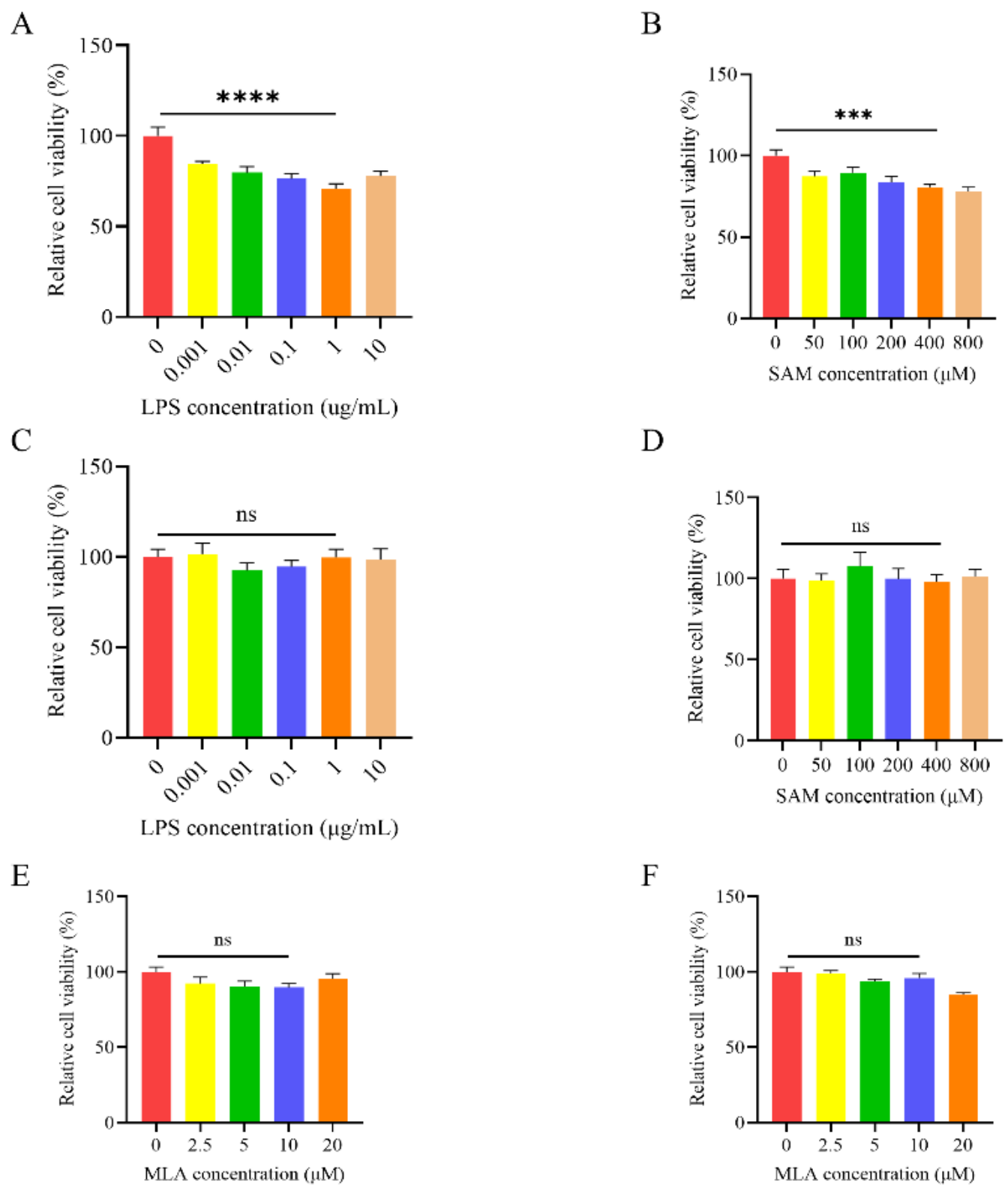

Supplementary Figure 6. CCK-8 assays for determining non-cytotoxic concentrations of LPS and SAM in BV2 and HT22 cells. (A, B) Effects of different concentrations of LPS and SAM on BV2 cell viability, as assessed by CCK-8 assay. (C, D) Effects of different concentrations of LPS and SAM on HT22 cell viability, as assessed by CCK-8 assay. (E) Effect of different concentrations of MLA on BV2 cell viability, as assessed by CCK-8 assay. (F) Effect of different concentrations of MLA on HT22 cell viability, as assessed by CCK-8 assay. Each concentration was tested in six technical replicate wells. Data are presented as mean ± SEM. Statistical significance is indicated as ns, $p \geq 0.05$; *** $p < 0.001$; **** $p < 0.0001$.
